# Collagenase Injection versus Limited Fasciectomy for Dupuytren’s Contracture

**DOI:** 10.1056/NEJMoa2312631

**Published:** 2024-10-09

**Authors:** Joseph Dias, Puvanendran Tharmanathan, Catherine Arundel, Charlie Welch, Qi Wu, Paul Leighton, Maria Armaou, Nick Johnson, Sophie James, John Cooke, Lionel Bainbridge, Michael Craigen, David Warwick, Samantha Brady, Lydia G. Flett, Judy Jones, Catherine N. Knowlson, Michelle Watson, Ada Keding, Catherine E. Hewitt, David Torgerson

**Affiliations:** 1 Academic Team of Musculoskeletal Surgery, Undercroft, Leicester General Hospital, University Hospitals of Leicester NHS Trust, Leicester, LE5 4PW, UK; 0116 258 4702; 2 York Trials Unit, Department of Health Sciences, University of York, York, YO10 5DD, UK; 3 Department of Health Sciences, University of York, York, YO10 5DD, UK; 4 University of Nottingham, Nottingham, NG7 2RD, UK; 5 University Hospitals of Leicester NHS Trust, Leicester, LE5 4PW, UK; 6 University Hospitals of Derby and Burton NHS Trust, Derby, DE22 3NE, UK; 7 Royal Orthopaedic Hospital NHS Foundation Trust, Birmingham, B31 2AP, UK; 8 University Hospital Southampton NHS Foundation Trust, Southampton, SO16 6YD, UK

## Abstract

**Background:**

Treatments for Dupuytren’s contracture include limited fasciectomy and collagenase injection. Evidence comparing these treatments is limited.

**Methods:**

We performed an unblinded, multicenter, pragmatic, two-arm, randomized controlled non-inferiority trial comparing collagenase injection to limited fasciectomy in persons with moderate Dupuytren’s contracture. The primary outcome was the score on the Patient Evaluation Measure Hand Health Questionnaire (range 0 to 100, higher scores indicating worse outcome) 1-year post-treatment. The pre-specified non-inferiority margin was 6 points.

**Results:**

672 participants were randomized (n=336 per group). Primary analysis included 599 (314 collagenase; 285 limited fasciectomy).

At 1-year, the mean score on the Hand Health questionnaire was 17.8 in the collagenase group (n = 284) and 11.9 in the limited fasciectomy group (n = 250); between group difference = 5.9 (95%CI: 3.1 to 8.8, one sided p value for non-inferiority 0.49). At 2 years (n = 229 and n = 197 in collagenase and limited fasciectomy groups, respectively), the between group difference was 7.2 (95%CI: 4.2 to 10.9). The percentage of persons with moderate to severe complications was 2% in the collagenase group and 5% in the limited fasciectomy group. Recurrent contracture requiring re-intervention occurred in 8% after collagenase versus 1.7% after limited fasciectomy.

**Conclusions:**

Collagenase was not non-inferior to limited fasciectomy with respect to the Patient Evaluation Measure hand health questionnaire score at 1-year post-treatment.

(Supported by NIHR Health Technology Assessment programme; Trial registration: ISRCTN18254597)

## Introduction

Dupuytren’s contracture is caused by a fibro-proliferative disease which forms fibrous cords in the hand.^1–3^ These cords progressively shorten, pulling one or more fingers into a bent position and increasingly interfere with hand function, potentially impairing quality of life. There is no cure for Dupuytren’s disease, and despite intervention, contractures may recur and need further treatment.

In Western countries, the prevalence of Dupuytren’s contracture ranges from 0.6 to 31.6% with higher prevalence in men, those over age 50 years, and those of Northern European descent.^4^ Smoking and occupations that involve manual labor or forceful stretching of the fascia are associated with increased risk of developing Dupuytren’s contracture.^4^

Limited fasciectomy is the most frequently used method to correct Dupuytren’s contracture in Europe and the USA.^5, 6, 7^ Another treatment involves injection of an enzyme, Collagenase clostridium histolyticum (Collagenase), into the cord, which weakens it by breaking down collagen; a few days after injection, the weakened cord is manipulated to rupture it and straighten the contracted joint. Collagenase was shown to be better than placebo in correcting the contracture to 0-5 degrees at 30 days.^8^ The benefits of collagenase treatment include its administration in a clinic instead of an operating room, potential cost savings^8–10^ and a quicker recovery time compared to limited fasciectomy.

The DISC trial compared the effectiveness and safety of collagenase injection to limited fasciectomy to treat moderate Dupuytren’s Contracture of ≥30°.^11^

## Methods

### Trial Design and Oversight

The DISC trial was a multicenter, open-label, pragmatic, parallel two-arm randomized controlled non-inferiority trial. The 31 recruiting sites were UK National Health Service hand units (Appendix S1). Data collection, monitoring and analysis were completed by the Academic Team of Musculoskeletal Surgery (University of Leicester, UK) and York Trials Unit (University of York, UK) with oversight from independent Trial Steering and Data Monitoring and Ethics Committees.

The study and amendments were approved by the Leeds West Research Ethics Committee, UK Health Research Authority and UK Medicines and Healthcare products Regulatory Authority. JD and CW take responsibility for the accuracy and completeness of reporting and for fidelity of the report to the study protocol.

### Population

Adult patients with a discrete, palpable Dupuytren’s cord causing contracture of ≥30°,^3, 8^ appropriate for both study treatments were eligible. Patients were excluded if they had: severe contractures (>135°); previous treatment on the study reference digit; pre- existing disorders affecting hand function or where participation would put them at risk ; a coagulation disorder; participated in another investigational medicinal product study within 12 weeks; or were pregnant or breastfeeding. ^12 13^ Participants provided written informed consent prior to randomization.

### Treatments

Collagenase was injected as three aliquots at set points following the current approved Summary of Product Characteristics.^10^ After 1-7 days, the cord was ruptured by manipulation in clinic under local anesthetic correcting the contracture.^8^

Limited fasciectomy was performed as day surgery and involved removal of diseased cords to correct the contracture. Surgery was followed by routine wound check appointments one to two weeks later.

### Outcomes

The primary endpoint was the Patient Evaluation Measure Hand Health Questionnaire score (range 0 to 100, higher scores indicating worse outcome) at 1 year post- treatment.^14^ The Patient Evaluation Measure was also completed at baseline, before treatment delivery, 3 months, 6 months, and 2 years post-treatment, with the additional post-treatment measurements serving as secondary endpoints.

Secondary outcomes included the Unité Rhumatologique des Affections de la Main scale^15^ (Range 0-45, higher scores indicate greater difficulties), Michigan Hand Questionnaire ^16, 17^ (Range 0-100, higher scores indicate better function, less pain, greater satisfaction), recurrence, extension deficit and total active movement, re- intervention (further treatment of the reference digit using Collagenase injection, limited fasciectomy, percutaneous needle fasciotomy or dermofasciectomy), time to function recovery (using a single assessment numeric evaluation measure),overall hand assessment and and complications relating to study treatments or to the reference hand, regardless of whether related to study treatment (Table S1 further describes all outcomes). We also collected information on other serious adverse events regardless of relation to study treatment.

Secondary patient reported outcomes were collected at baseline, and the same post- treatment time points as the Patient Evaluation Measure, except for Single Assessment Numeric Evaluation (also collected at 2- and 6-weeks) and the Michigan Hand Questionnaire (only collected at 1 and 2-years). Outcomes were collected in hospital clinics, with some participants followed up by post, telephone or video-call during the COVID 19 pandemic.^18^

### Randomization and Blinding

Participants were randomized 1:1 to receive collagenase injection or limited fasciectomy, using randomly varying block sizes (Sizes 4 and 6) and stratification by the reference joint (metacarpophalangeal or proximal interphalangeal joint). The randomization sequence was amended, from 21^st^ January 2020, to include stratification by center.

The allocation sequence was generated by the trial statistician, independent of the recruiting sites, and a secure central online randomization service (Sealed Envelope Ltd) was used.

Clinicians and participants could not be blinded due to the nature of trial interventions. The analyzing statistician (CW) was not blinded when performing the final analyses according to the pre-specified analysis plan.

### Sample Size

Previous survey data suggested a Dupuytren’s contracture population standard deviation of ~22 points Patient Evaluation Measure Hand Health Scores.^19^ In the absence of published consensus regarding an appropriate non-inferiority margin, we used previous data collected pre- and post-treatment and an anchor question regarding overall hand function to estimate the mean difference in Patient Evaluation Measure score associated with a minimal change in qualitative assessment of hand function. We assumed that this difference in score (6 points) represents the minimum threshold at which changes in hand function become appreciated by patients.

Using these parameters, a sample size of 568 participants (284 per arm) was required to obtain 90% power to test *H*_0_: *δ* ≥ 6 vs *H*_1_: *δ* < 6 with one sided p < 0.025. Assuming 20% attrition at the 1-year follow-up, the total target sample size was 710.

### Data Analysis

A statistical analysis plan was approved by the independent data monitoring committee, prior to completion of recruitment.^20^ All analyses were undertaken at the end of the follow-up period using Stata/SE-v17.0. For all outcomes, the unit of analysis was the patient (rather than digits or joints). Where participants had multiple joints affected, a single joint was selected before randomization as the study reference joint, with analyses being based on the measurements obtained for the designated digit and joint.

Baseline data were summarized by allocation. A Consolidated Standards of Reporting Trials (CONSORT) diagram summarized participant flow and data completeness.^21^ No adjustment for multiple outcomes was pre-specified; hence a p value is reported only for the primary endpoint. For secondary outcomes, 95% confidence intervals were not adjusted for multiplicity.

For the primary analysis, a longitudinal model, incorporating all available post-treatment Patient Evaluation Measure scores, was used to estimate differences in scores at each timepoint. All participants with at least one post-treatment measurement available were included in this analysis as part of their allocated treatment group in accordance with the statistical analysis plan. Treatment group, timepoint and their interaction were included as fixed effects, along with study reference joint type (the stratification factor), baseline primary outcome score and a random intercept for center. An unstructured covariance matrix modelled correlation between repeated measurements.

The null hypothesis that collagenase is inferior to limited fasciectomy was rejected if the upper bound of the two-sided 95%confidence interval for the difference at 1 year was less than the non-inferiority margin of six points. Pre-specified sensitivity analyses investigated the robustness of the primary analysis results to departures from the planned timing of treatment delivery and follow-up assessments, and different assumptions about the missing outcome data, including several multiply imputed data analyses including all randomized participants.

To assess the potential impact of non-adherence to the allocated treatment, we used an instrumental variable estimator (with random allocation as the instrument) to estimate the Complier Average Causal Effect at 1 year (Appendix S2).^22, 23^

Two subgroup analyses investigated variation in treatment effects across subgroups associated with baseline characteristics, one prespecified analysis according to baseline treatment preference (preferred collagenase, preferred limited fasciectomy, or no preference) and one post-hoc analysis according to study reference joint (metacarpophalangeal or proximal interphalangeal joint).

Continuous secondary outcomes were analyzed in a manner similar to the primary outcome. Categorical secondary outcomes were analyzed using appropriate binary or ordinal logistic regression models, with treatment effects reported as odds ratios or risk difference. A Cox proportional hazards model estimated the relative hazard of re- intervention to the reference digit and the absolute differences in risk of re-intervention by 2 years.

## Results

### Patient Characteristics

Participants were recruited from 31^st^ July 2017 to 28^th^ September 2021, with follow up planned for a minimum of 1 year from treatment. We randomized 672 participants, with 336 allocated to collagenase injection and 336 to limited fasciectomy. This was below the target sample size of 710, but larger than the effective sample size of 568 targeted for the primary analysis (Appendix S3). Baseline characteristics were similar between groups (Table 1).

### Treatment Delivery and Follow Up

Of the 672 randomized participants, 621 (92.4%) received treatment as part of the trial, 27 (4.0%) withdrew consent before treatment delivery, and 24 (3.6%) did not receive treatment before the end of the data collection period. Treatments were delivered by surgeons routinely providing these interventions (Table S2).

Cross-over was limited: one participant (0.3%) allocated to collagenase received limited fasciectomy; seven participants allocated to limited fasciectomy received collagenase (2.1%). On average participants received collagenase within 8 weeks of randomization (Interquartile Range 4.6 to 12.6) and limited fasciectomy within 12.1 weeks (Interquartile Range 8.3 to 20.4). Most had one digit treated (Collagenase n=315 95.2 %, Limited fasciectomy n=237 82.6%). Treatment achieved full correction (≤5° extension deficit assessed immediately following manipulation or after surgery) in 92.0% of collagenase participants and 96.5% of limited fasciectomy participants.

**Figure 1:**
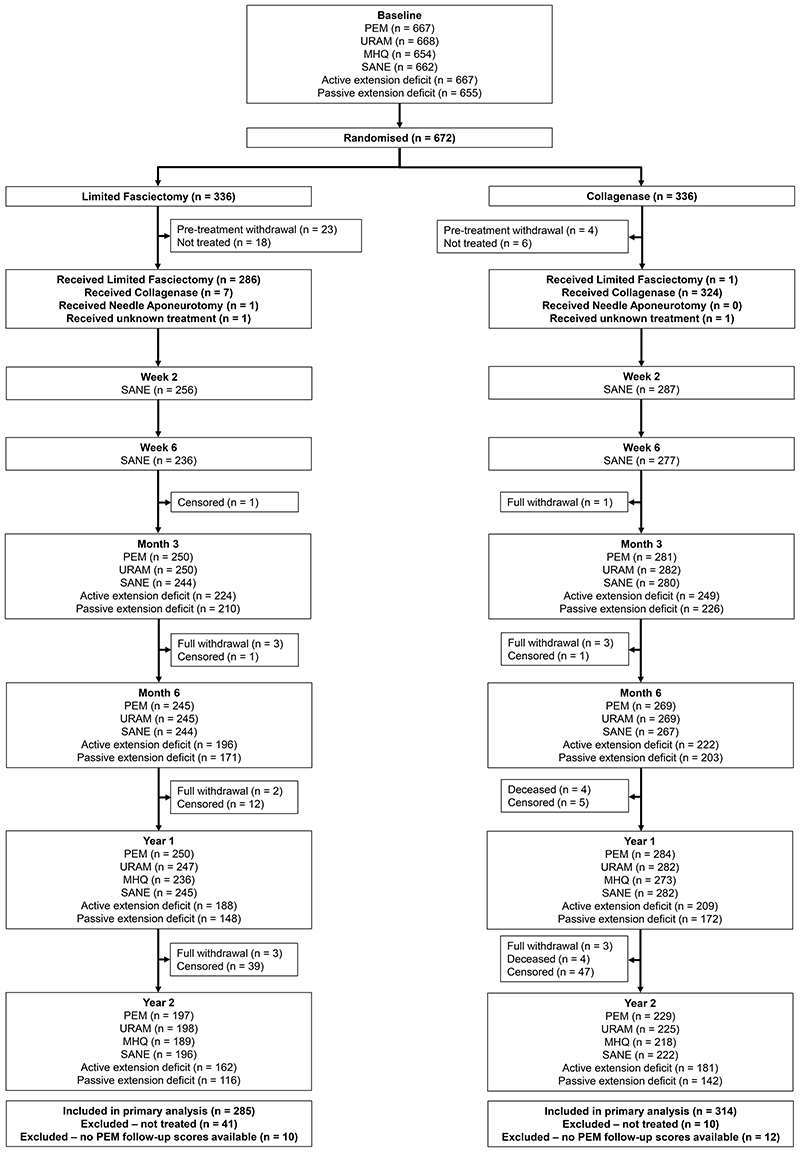
Participant flow diagram. Participants are categorized as censored if the trial follow-up period finished prior to the follow-up time point being due/completed

Of the 621 treated participants (326 in the collagenase group and 295 in the limited fasciectomy group), 599 (96.5%) provided primary outcome data that contributed to the primary analysis, 534 (86.0%) provided primary endpoint data at 1 year and 426 (68.6%) provided primary PEM scores at 2 years. Figure 1 details treatment delivery and follow-up data collection.

### Patient Reported Outcomes

**Figure 2:**
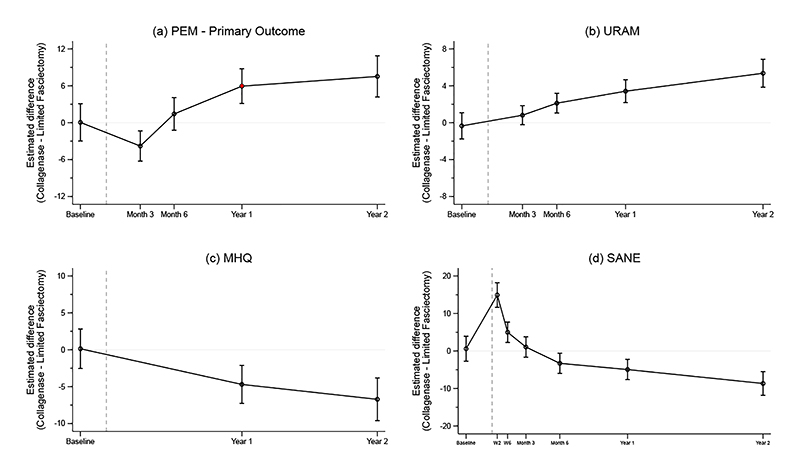
Estimated differences in expected scores and two-sided 95% confidence intervals over time for the four participant reported outcome measures. The vertical grey dashed line is plotted at the median time elapsed between baseline and treatment delivery, with subsequent time points referenced to this. For plots (a) and (b), values above zero indicate greater benefit from LF. For plots (c) and (d), values above zero indicate greater benefit from collagenase. 95% CIs are not adjusted for multiplicity and should therefore not be used for hypothesis testing with respect to any of the secondary endpoints. Figure 2a: Patient Evaluation Measure. The estimated differences at each time point are: 3 months = -3.8 (95%CI -6.2 to -1.3), 6 months = 1.4 (-1.2 to 4.1), 1 year = 5.9 (3.1 to 8.8), 2 years = 7.5 (4.2 to 10.9). The non-inferiority margin for the primary endpoint (Patient Evaluation Measure at 1 year) was +6 points. Figure 2b: Unité Rhumatologique des Affections de la Main scores (numerical estimates provided in supplementary material) Figure 2c: Michigan Hand Questionnaire scores (numerical estimates provided in supplementary material) Figure 2d: Single Assessment Numeric Evaluation scores (numerical estimates provided in supplementary material)

At 1-year, the Patient Evaluation Measure Hand Health Questionnaire score was 17.8 in the collagenase group and 11.9 in the limited fasciectomy group (estimated difference based on primary analysis model: 5.9, 95%CI 3.1 to 8.8, p, non-inferiority 0.49), indicating that collagenase was not non-inferior to limited fasciectomy. Results at 2 years were consistent with the primary analysis (between group difference 7.2, 95%CI: 4.2 to 10.9). Treatment effects at each timepoint are shown in Figures 2 and S1. Results were similar when using multiply imputed data (difference at 1 year = 6.5, 95% CI 3.9 to 9.2) and in other pre-specified and post-hoc sensitivity analyses (Tables S3-S9 and Figures S2-S4) including a post-hoc per-protocol analysis (difference at 1 year = 6.4, 95%CI 3.6 to 9.2). The estimate of the Complier Average Causal Effect at 12 months was 5.30 (95%CI 2.9 to 7.7), also consistent with results of the primary analysis. There was no substantial evidence of variation in treatment effects according to subgroup (Appendix Tables S10 − S13 and Figures S5 and S6).

Figure 2 and Table S14 show results for other patient reported outcome measures (Unité Rhumatologique des Affections de la Main, Michigan Hand Questionnaire and Single Assessment Numeric Evaluation). These results appeared generally consistent with those for the primary outcome.

At 1-year, 88.1% of participants treated with limited fasciectomy reported that overall their hand was “cured” or “much better”, compared to 68.6% of participants treated with collagenase (estimated marginal risk difference from planned analysis model = -17.1%, 95%CI -22.3% to -11.9%).

### Joint Measurements

**Figure 3:**
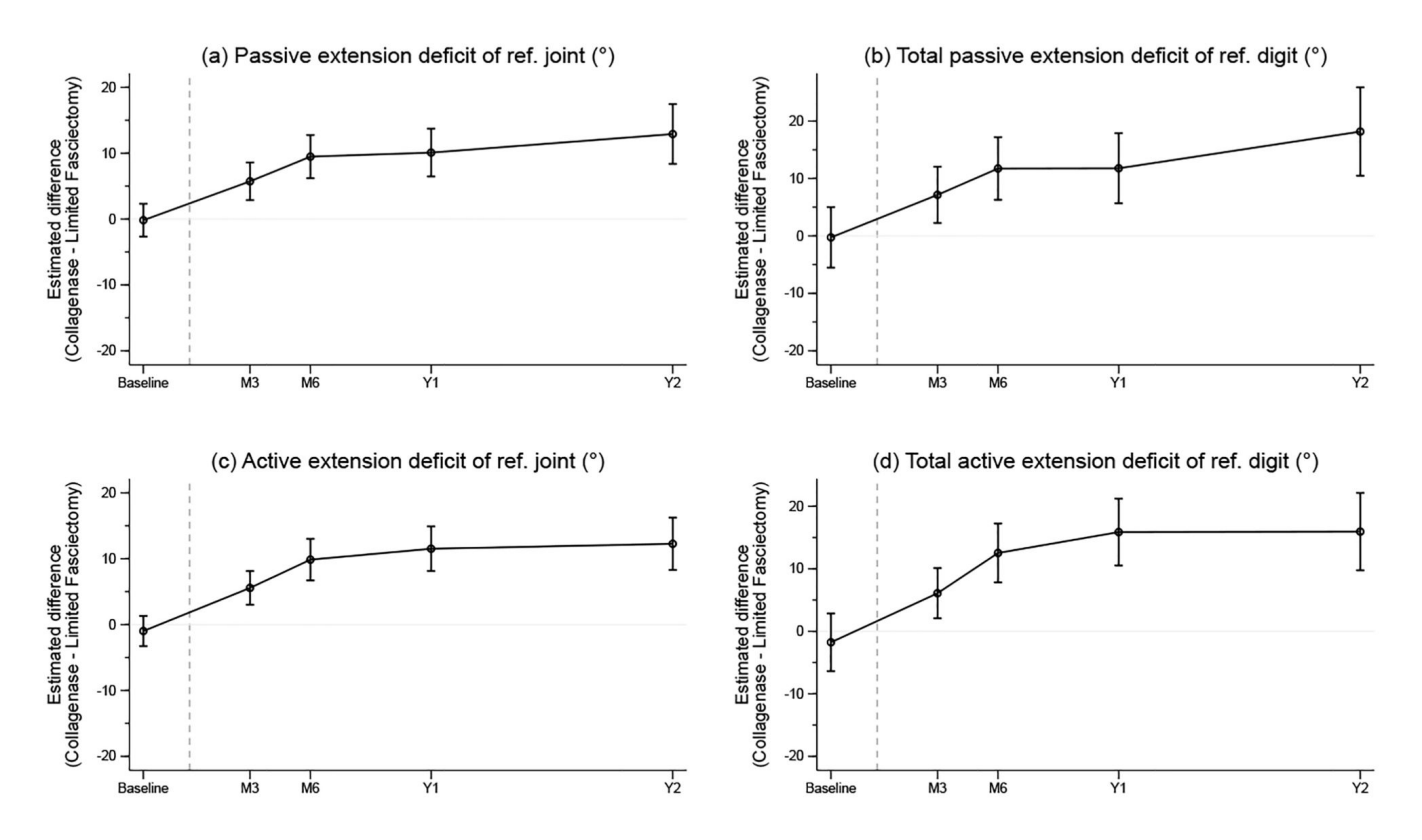
Estimated differences in passive and active extension deficit. The vertical grey dashed line is plotted at the median time elapsed between baseline and treatment delivery, with subsequent time points referenced to this. For all plots, values above zero indicate greater benefit from limited fasciectomy. Measurements were carried out by trained surgeons, therapists or research nurses using a goniometer and supported by a goniometry measurement manual. The interval estimates for these secondary endpoints have not been adjusted for multiplicity and should not be used for hypothesis testing.

Following treatment, the between group difference in passive extension deficit of the reference joint ranged from 5.7° (95%CI 2.9 to 8.6) at 3 months to 10.1° (95%CI 6.7 to 13.7) at 1 year (favoring the limited fasciectomy group) and increasing up to 2 years (Figures 3a and S7). Results for total passive extension deficit of the reference digit were similar (Figure 3b and S7). Among participants with available recurrence data, 32/186 (17.2%) patients in the collagenase group and 22/159 (13.8%) participants in the limited fasciectomy group had recurrence of contracture (estimated risk difference 4.0% 95%CI -3.7% to 11.7%). Detailed summaries of joint measurement outcomes are available in Table S15.

The between group difference in active extension deficit of the reference joint ranged from 5.6° (95%CI 3.0 to 8.1) at 3 months to 11.5° (95%CI 8.1 to 14.9) at 1 year (also favoring the limited fasciectomy group) and increasing up to 2 years (Figure 3c and S7). Results for total active extension deficit were similar (Figure 3d and S7).

Assessments based on multiply imputed data yielded similar results for all joint measurement outcomes.

### Complications and Re-intervention

There was no substantial difference between groups in the overall frequency of complications, but the collagenase group experienced fewer moderate or severe procedural complications than the limited fasciectomy group (2% vs 5%; risk ratio 0.4 95%CI 0.1 to 0.9). During up to 2 year follow-up, there were 8 deaths in the collagenase group and none in the limited fasciectomy group; all occurred more than 6 months after the procedure, and causes of death were unrelated to the procedure. Table 2 and Table S16 provide details of the frequency and severity of complications. (Table S17 details moderate and severe complications).

Of the 621 participants treated as part of the trial, 327 (52.7%) had complete data on re-interventions up to two years. Of these, 26/178 (8.0%) patients in the collagenase group and 5/149 (1.7%) participants in the limited fasciectomy group had re-intervention using collagenase, limited fasciectomy, percutaneous needle fasciotomy or dermofasciectomy. Comparing collagenase to limited fasciectomy, the hazard ratio for time to first re-intervention was 4.7 (95%CI 1.8 to 12.3).

## Discussion

In this multicenter randomized trial, collagenase was not non-inferior to limited fasciectomy for Patient Evaluation Measure Hand Health Questionnaire score at 1-year post-treatment. Results appeared similar between groups for other patient reported outcomes and for finger joint contracture at 1 year.

While results for the primary outcome and other patient-reported outcomes at early follow-up appeared to favor the collagenase group, this apparent advantage was not sustained. For all patient reported and joint contracture outcomes, the differences between groups, favoring limited fasciectomy, increased over time. Previous follow up data after collagenase injection showed increasing numbers of recurrences over time.^9, 24^

Surgical treatment permits correction of multiple affected joints and fingers whereas collagenase injection was restricted to up to two injections at a time. This could contribute to greater improvement in function after limited fasciectomy.

Based on the definition of recurrence used in this study, there was little evidence at 1 year that recurrence was more common after collagenase, despite limited fasciectomy providing greater improvement in passive and active extension deficit.

Re-intervention rates in the first 2 years after initial correction appeared to favor limited fasciectomy; eight percent of patients who were followed for 2 years in the collagenase group had re-intervention compared to 1.7% of the limited fasciectomy group.

The percentage of persons in the collagenase and limited fasciectomy groups who experienced complications overall was similar between groups and consistent with previously reported complication rates.^8, 12^ Most complications were classified as mild, such as pain at the injection site, skin tears or swelling, which resolve quickly with minimal or no treatment. However, the proportion of participants with moderate or severe complications was higher in the limited fasciectomy group (5% vs 2% respectively).

Limitations of our study should be noted. Substantive changes to provision of care for patients with Dupuytren’s Contracture impacted treatment delivery at different points during the trial, including changes to prioritization and delivery processes in specific localities and suspension of treatment and follow-up during the COVID-19 pandemic. Roughly 31% of treated participants could not complete 2 year follow-up within the funded period^18^ due to pandemic related delays. Therefore, some recurrences and reinterventions may not have been captured. Longer term follow-up is needed to better assess progression of contracture and re-intervention rates over time. Almost all participants were White (Table S18), potentially limiting generalizability, but Dupuytren’s Disease has been reported to predominantly affect Causasian persons of North Western European descent (Table S18). Despite our findings favoring limited fasciectomy, collagenase injection may be preferred by patients who prioritize avoiding the higher risk of moderate to severe complications and longer time to improvement associated with surgery.

In this multicenter randomized trial, collagenase was not non-inferior to limited fasciectomy with respect to the Patient Evaluation Measure hand health questionnaire score at 1-year post-treatment. Moderate or severe adverse events were uncommon but more frequent in the limited fasciectomy group.

## Supplementary Material

Supplement

## Figures and Tables

**Table 1:** Baseline characteristics of randomized participants*

	Limited Fasciectomy N = 336	Collagenase N = 336
**Age (years)**	66.5 (9.2)	66.4 (8.8)
**Male, n (%)**	263 (78.3)	270 (80.4)
**Ethnicity**		
White	333 (99.1)	332 (98.8)
Mixed Race	1 (0.3)	1 (0.3)
Asian/Asian British	1 (0.3)	3 (0.9)
Missing	1 (0.3)	0 (0.0)
**History of bilateral disease, n (%)**	170 (50.6)	174 (51.8)
Missing	15 (4.5)	11 (3.3)
**Family history of Dupuytren’s disease, n (%)**	126 (37.5)	112 (33.3)
Missing	1 (0.3)	3 (0.9)
**History of Garrods pads, n (%)** ^b^	54 (16.1)	43 (12.8)
Missing	58 (17.3)	55 (16.4)
**History of Peyronie’s disease, n (%)** ^b^	11 (3.3)	14 (4.2)
Missing	44 (13.1)	44 (13.1)
**History of Ledderhose disease, n (%)** ^b^	24 (7.1)	18 (5.4)
Missing	61 (18.2)	56 (16.7)
**Study reference digit, n (%)**		
Thumb	1 (0.3)	1 (0.3)
Index	4 (1.2)	0 (0.0)
Middle	24 (7.1)	17 (5.1)
Ring	109 (32.4)	111 (33.0)
Little	198 (58.9)	207 (61.6)
**Study reference joint, n (%)**		
Metacarpophalangeal	207 (61.6)	221 (65.8)
Proximal interphalangeal joint	129 (38.4)	115 (34.2)
**Active extension deficit of ref. joint (°)**	52.3 (15.4)	51.5 (16.7)
**Passive extension deficit of ref. joint (°)**	45.9 (16.4)	45.8 (17.3)
**Patient Evaluation Measure Hand Health Questionnaire** ^a^	34.1 (19.7)	34.2 (20.2)
**Unité Rhumatologique des Affections de la Main total score** ^b^	17.2 (9.5)	17.0 (9.3)
**Michigan Hand Questionnaire total score** ^c^	67.5 (17.7)	67.6 (17.1)

a Range 0 - 100, higher scores indicate greater disability;

b Range 0 - 45, higher scores indicate greater difficulties;

c Range 0 - 100, higher scores indicate better function, less pain, greater satisfaction. *

b These conditions are associated with Dupuytren’s contracture. Ledderhose’s disease is plantar fibromatosis creating slow-growing nodules usually in the plantar fascia of the instep and rarely causing flexion contractures of toes. Garrod’s pads/knuckle pads are fibrous pads with myofibroblast proliferation almost always occurring over the dorsum of one or more proximal interplalangeal finger joints. Peyronie’s disease is fibromatosis creating a plaque in part of the penis which can cause the penis to bend, especially during erection.

**Table 2:** Complications and adverse events^ǂ^ according to treatment allocation.

Classification of complications/adverse eventsǂ~		
	Limited fasciectomy	Collagenase
	N = 295	N = 326
Pain, swelling or stiffness, n (%)*	48 (16.3)	70 (21.5)
Skin, scar or wound related (excluding wound infection), n (%)*	36 (12.2)	59 (18.1)
Wound (infection), n (%*)^~^	6 (2.0)	0 (0.0)
Nerve related, n (%*)	42 (14.2)	18 (5.5)
Circulation or bleeding related, n (%*)	13 (4.4)	46 (14.1)
Raynauds, n (%*)	5 (1.7)	2 (0.6)
Amputation, n (%*)	1 (0.3)	0 (0.0)
Complex Regional Pain Syndrome - algodystrophy, n (%*)	2 (0.7)	0 (0.0)
Lymph related	0 (0.0)	5 (1.5)
Other, n (%*)^¶^	4 (1.4)	6 (1.8)
Instability, n (%*)	0 (0.0)	1 (0.3)
**Complication Severity** **		
	**Limited** **Fasciectomy** N = 295	**Collagenase** N = 326
None, n (%*)	189 (64.1)	191 (58.6)
Very minor, n (%*)	20 (6.8)	58 (17.8)
Mild, n (%*)	90 (30.5)	105 (32.2)
Moderate, n (%*)^~^	13 (4.4)	6 (1.8)
Severe, n (%*)	3 (1.0)	0 (0.0)
**Other Serious Adverse Events** ǂ	**Limited** **fasciectomy**	**Collagenase**
	n=336	n=336
Death (n, %)	0 (0.0)	8 (2.4)^

ǂ Complications and hand specific events including those not related to treatment were considered adverse events.

~ One of the wound infections was an abscess on another digit considerered possibly related to treatmen’ (moderate severity).

* Of total number of participants (by arm or overall).

** Worst severity of treatment complication experienced following trial treatment, ranked using the following seven level scale: None, Very minor, Mild, Moderate, Severe, Devastating, Death. No procedural complications or hand -spec events were classified as devastating or led to death.

^ Deaths: None of the 8 deaths were considered related to the study reference digit or treatments. Causes of death included: COVID 19 (n=2) at 7 and 11 months after study treatment; complications following unrelated surgery (n=2) (major abdominal surgery and death 20 months after study treatment, and following discharge from surgery to place stents after myocardial infarction 11 months after study treatment); lung cancer (n=1) 20 months after treatment;; pre-existing medical condition (details of condition unavailable; death 7 months after study treatment) (n=1); brain hemorrhage (n=1); cause not provided, death 17 months after study treatment (n=1).

¶ Other events for collagenase patients: Cubital tunnel (n=3); Dizziness (n=1); Headache (n=1); Nausea (n=1);Pruritis (n=3). Other events for Limited fasciectomy patients: Delayed discharge (n=1); Carpal tunnel (n=1), Cubital tunnel (n=1); Dizziness (n=1); Pruritis (n=1).
