# Supplementary material for: Collagenase Injection versus Limited Fasciectomy for Dupuytren’s Contracture
Source: N Engl J Med. Author manuscript; Available in PMC 2024 Oct 24. (PMC7616701; doi:10.1056/NEJMoa2312631)
Supplement: Supplement [file EMS197674-supplement-Supplement.pdf]

Collagenase injection versus limited fasciectomy for Dupuytren's Contracture.

Appendices Contents

Participating Study Sites and Principal Investigators	Pages 3-4
Sensitivity Analyses for the Primary Outcome – Methods	Pages 5-7
Subgroup Analyses - Methods	Page 7
Discussion of Sample Size Shortfall	Page 7
Figure S1: Mean score profiles over time stratified by treatment group for the patient reported outcome measures	Page 8
Figure S2: PEM Hand Health Questionnaire scores at 12 months post-treatment by allocation	Page 8
Figure S3: Estimates of difference in expected PEM score at 12 months post-treatment under various informative departures from MAR	Page 9
Figure S4: Point estimates and pointwise two-sided 95% confidence band for the difference in expected PEM score	Page 10
Figure S5: Point estimates and two-sided 95% confidence intervals for the difference (Collagenase - LF) in expected PEM score by baseline treatment preference subgroup and time point	Page 10
Figure S6: Point estimates and two-sided 95% confidence intervals for the difference (Collagenase - LF) in expected PEM score by study reference joint subgroup and time point	Page 11
Figure S7: Mean score profiles over time stratified by treatment group for the joint measurement outcomes	Page 11
Table S1: DISC Outcomes and results summary table	Pages 12-19
Table S2: Experience of surgeons delivering/supervising collagenase administration and limited fasciectomy surgery	Page 20
Table S3: Treatment effect estimates at each post-treatment time point obtained from univariate semi-parametric analyses (proportional odds model) of the available scores	Page 20
Table S4: Treatment effect estimates at 3, 6, 12 and 24 months post-treatment obtained from primary analysis model including additional baseline predictors of incomplete primary outcome data	Page 20

Table S5: Treatment effect estimates at 3, 6, 12 and 24 months post-treatment from primary analysis undertaken using multiply imputed data	Page 21
Table S6: Treatment effect estimates at 3, 6, 12 and 24 months post-treatment obtained from an analysis of the primary outcome data conditioning on the pre-treatment PEM measurements	Page 21
Table S7: Treatment effect estimates at 3, 6, 12 and 24 months post-treatment obtained from an analysis including only primary outcome data collected within the protocol specified follow-up windows	Page 21
Table S8: Treatment effect estimates at 3, 6, 12 and 24 months post-treatment obtained from analysis including (variable) time from treatment as a continuous predictor	Page 22
Table S9: Results from post-hoc per-protocol analysis requested during review (boldface denotes the primary endpoint)	Page 22
Table S10: Baseline treatment preferences – participants with available primary outcome data only	Page 22
Table S11: Designated study reference digit – participants with available primary outcome data only	Page 23
Table S12: Treatment effect estimates by baseline treatment preference subgroup and time point (includes only participants with non-missing baseline treatment preferences	Page 23
Table S13: Treatment effect estimates by baseline reference joint subgroup and time point	Page 23
Table S14: Summaries of patient reported outcome scores by time point and allocation (all available cases)	Page 24-26
Table S15: Summaries of joint measurement outcomes by time point and allocation (all available cases)	Page 26-29
Table S16: Details of treatment complications and adverse events‡ according to treatment allocation.	Page 29-30
Table S17: Details of treatment complications graded as “Severe” or “Moderate” in severity.	Page 31
Table S18: Representativeness of DISC study population	Page 32
References	Page 34

Appendix S1: DISC Participating Sites and Principal Investigators

Basingstoke and North Hampshire Hospital *(Mr J Hobby; Mr D Avis)*

Bedford Hospital *(Mr J Malal)*

Royal Blackburn Hospital *(Mr F Younis)*

Chelsea and Westminster Hospital *(Ms A Bremner Smith)*

City Hospitals Sunderland *(Ms S Kettle-Klenka; Mr D Dowen)*

Derriford Hospital - Plymouth *(Ms S Fullilove)*

Glenfield Hospital - Leicester *(Mr B Bhowal; Prof' J Dias)*

Gloucestershire Royal Hospital *(Mr W Mason)*

James Cook University Hospital - South Tees *(Mr C Coapes)*

King's College Hospital *(Mr K Karuppaiah)*

Lister Hospital *(Mr H Ridha)*

Northern General Hospital – Sheffield *(Ms G Rose)*

Princess Royal Hospital – Brighton *(Ms R Johnson)*

Queen Alexandra Hospital – Portsmouth *(Mr P Sauve)*

Robert Jones and Agnes Hunt Orthopaedic Hospital *(Mr C Kelly)*

Royal Bolton Hospital *(Mr J Wilson)*

Royal Cornwall Hospital *(Ms R Dunlop)*

Royal Derby Hospital *(Prof LC Bainbridge)*

Royal Liverpool and Broadgreen Hospital *(Mr D Brown)*

Royal Orthopaedic Hospital – Birmingham *(Mr M Craigen)*

Royal Victoria Infirmary – Newcastle *(Mr R Milner; Ms S Stevenson)*

Russell's Hall Hospital – Dudley *(Mr N Sarhadi; Mr H Bella)*

University Hospitals Southampton *(Prof’ D Warwick)*

St Georges Hospital – London *(Ms S Umarji)*

University Hospital of Coventry and Warwickshire *(Mr A Mahon)*

University Hospital Monklands *(Ms S Phillips)*

University College Hospital *(Ms A Davy)*

University Hospital of North Tees *(Mr L Ajekigbe)*

Warrington and Halton Hospital *(Mr N Shah)*

Western General Hospital – Edinburgh *(Mr N Cahoon)*

Wrightington Hospital *(Mr S Talwalkar)*

Appendix S2: Sensitivity analyses for the primary outcome

Distributional assumptions

Summaries of the available PEM HHQ scores (the primary outcome) at each time point are given in Table S3. The marginal distributions of the PEM scores at 12 months (the primary endpoint) in each group are shown in Figure S3. In general, the scores at all follow-up time points are towards the lower (i.e. better) end of the PEM scoring scale, with approximately 75% of patients having scores between 0 and 20 (out of 100) at each post-treatment time point and over 25% of available cases in the LF group having scores of zero at 12 and 24 months. This abundance of scores at or near zero is evident as skew in the marginal distribution of the month 12 scores in Figure S3 and is indicative of poor discrimination of different levels of Dupuytren's related disability at this more favourable end of the scale (i.e. a ceiling effect).

The ceiling effects noted mean the primary outcome data show some departure from the assumptions of the planned primary analysis. Table S5 presents the estimates and relevant tests from a series of univariate semi-parametric analyses that are more robust to the ceiling effects observed. These analyses were specified as contingencies in the Statistical Analysis Plan.

Missing data assumptions

The primary analysis is valid under the assumption that any missing primary outcome data (at any post-treatment follow-up) are missing at random (MAR) with respect to the predictors included in the analysis model (i.e. allocation, study reference joint type, baseline score and recruitment site) and any observed primary outcome data. Tables S6 and S7 report the results from several pre-specified analyses to investigate the sensitivity of results to variation in, and departures from, the MAR assumption outlined above.

To investigate the sensitivity of the primary analysis to various systematic departures from MAR at 12 months, we undertook a delta-based sensitivity analysis using a mean score and pattern mixture modelling approach. The estimated differences in PEM score at 12 months, under different departures from MAR (i.e. $\delta = \mathbb{E}[Y \mid X, \text{Missing}] - \mathbb{E}[Y \mid X, \text{Not missing}]$) are illustrated in Figure S4. All missing not at random (MNAR) scenarios considered assume that missingness is associated with poorer (i.e. higher) responses for the primary outcome (conditional on the covariates in the substantive analysis – reference joint and baseline PEM score).

Departures from the planned treatment and follow-up schedule

We undertook several pre-specified analyses of the primary outcome to investigate the sensitivity of the primary analysis results to differences in time to treatment between groups and departures from the planned follow-up schedule.

We repeated the primary analysis conditioning on the pre-treatment measurements in place of the baseline measurements (with all other aspects of the analyses being kept the same). This analysis included the same 599 participants as were included in the primary analysis (285 LF and 314 collagenase). The results of this analysis are reported in Table S8.

To assess the sensitivity of the results of the primary analysis to departures from the planned follow-up schedule, we undertook two additional analyses of the primary outcome data, one pre-specified and one post-hoc. The pre-specified analysis was to repeat the primary analysis including only primary outcome data collected within the protocol specified windows for completion. The results of this analysis are reported in Table S9. The post-hoc analysis aimed to directly model the effects of time from treatment using generalised least squares (with a random intercept for recruitment site). This analysis modelled the effects of time from treatment using a four knot restricted cubic spline (knots placed at 3, 6 12 and 24 months), and within patient correlation using an exponential covariance structure for the residual errors. This model was used to derive point and interval estimates at 3, 6, 12 and 24 months post-treatment to facilitate comparison with the results of the primary analysis (see Table S10), as well as estimates of the treatment effect over all time points up to 27 months post-treatment (see Figure S5).

Per-protocol analyses

The primary analysis reported in the main text includes all treated participants with primary outcome data available for at least one post-treatment follow-up time point. This is in line with the pre-specified analysis plans.

Upon request of the deputy editor we undertook a post-hoc per-protocol analysis including only participants that received their allocated treatment. The results of this analysis are shown in Table S11.

CACE analysis

An instrumental variable estimator (with random allocation as the instrument) was used to estimate the Complier Average Causal Effect at 1 year, that is the causal effect of treatment among the partially latent subgroup of participants that would have received collagenase if and only if they were allocated collagenase.

For all participants that received treatment (and therefore could feasibly have follow-up data) compliance was defined in a binary manner. Participants that received a treatment different from the one allocated were assigned to a “non-complier” principal stratum for the purposes of estimating the complier average causal effect.

The estimate of the complier average causal effect at 1 year was 5.3 (95%CI 2.9 to 7.7, confidence interval not subject to any adjustment for multiplicity).

Subgroup analyses

We undertook two subgroup analyses to investigate the presence and extent of treatment effect heterogeneity associated with baseline treatment preference (preferred collagenase, preferred LF, no preference), and designated study reference joint (MCP or PIP). Brief summaries of these baseline variables for the participants with primary outcome data for at least one post-treatment time point are given in Tables S13 and S14. Treatment effects by subgroup and time point were estimated via addition of; the main effects of the subgroup variable to the primary analysis model (if not already present), the two-way interactions between subgroup and treatment group, and the three-way interactions between subgroup, treatment group and time point. Estimates by preference subgroup (and time point) are given in Table S15 and Figure S6. Estimates by study reference joint subgroup (and time point) are given in Table S16 **Error! Reference source not found.**and Figure S7.

Appendix S3: Discussion of Sample Size Shortfall

Firstly, the figure of 710 was based on an effective sample size of 568 and 20% attrition by 12 months post-treatment (the primary endpoint). The primary analysis model included 534 (94% of target effective sample size) PEM scores from 12 months post-treatment and included PEM scores for at least one post-treatment time point from a total of 599 participants (89% of participants randomised). Hence the shortfall for the planned effective sample size was relatively modest. The additional precision that would have been gained from an additional 34 available primary endpoints would be very unlikely to have made the upper limit of the two-sided 95% confidence interval for the difference at 12 months less than 6, and is therefore very unlikely to have changed to conclusions of the primary analysis.

Furthermore, the target effective sample size was based on the number of patients required to obtain 90% power for a t-test comparing the 12 month scores between groups (under the assumptions specified in the “Sample Size” sub-section of the Methods section). However, the pre-specified primary analysis was based on a longitudinal model that incorporated all post-treatment measurements as outcomes and conditioned on informative baseline covariates. Both aspects will have increased the precision of the primary analysis compared with the more conservative analysis assumed for the sake of the power calculations/analysis.

Figure S1: Mean score profiles over time stratified by treatment group for the patient reported outcomes. These estimates are based on the expected scores obtained from the pre-specified analysis models for each outcome. Unadjusted summary statistics are shown for each outcome at each time point in Table S3

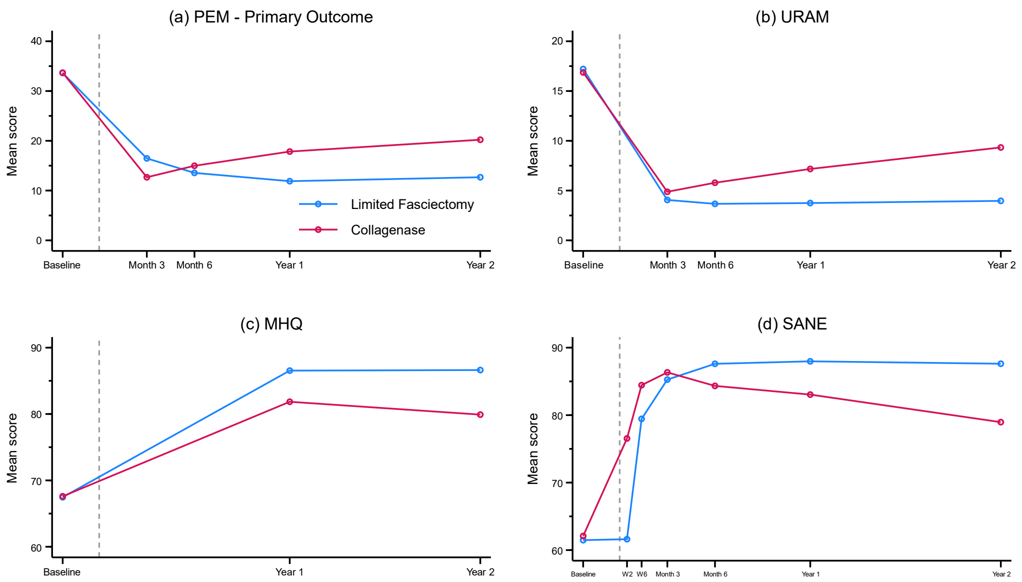

Figure S2: PEM Hand Health Questionnaire scores at 12 months post-treatment by allocation (kernel density estimates, available cases only)

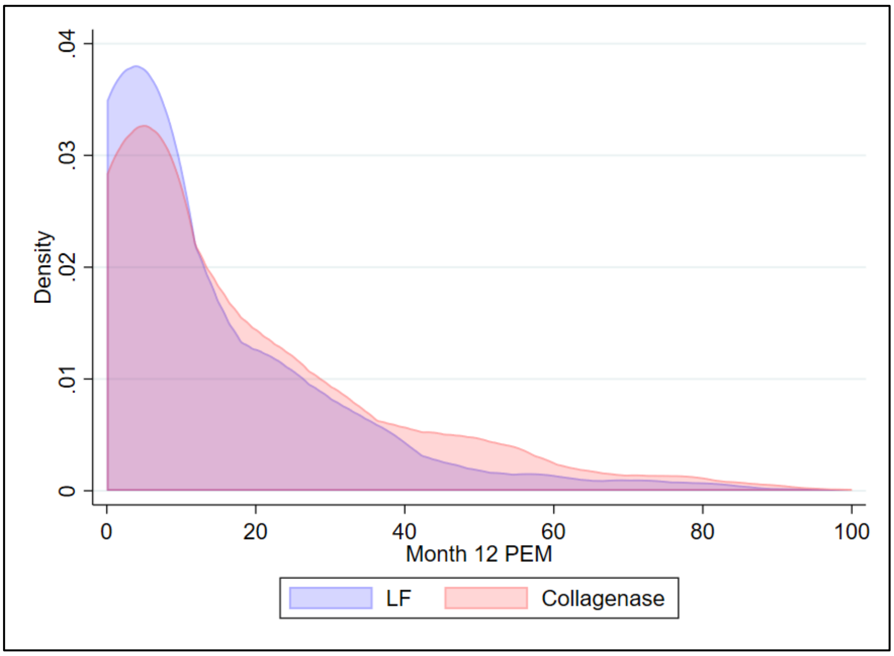

Figure S3: Estimates of difference in expected PEM score at 12 months post-treatment under various informative departures from MAR. The 95% confidence intervals are not adjusted for multiplicity.

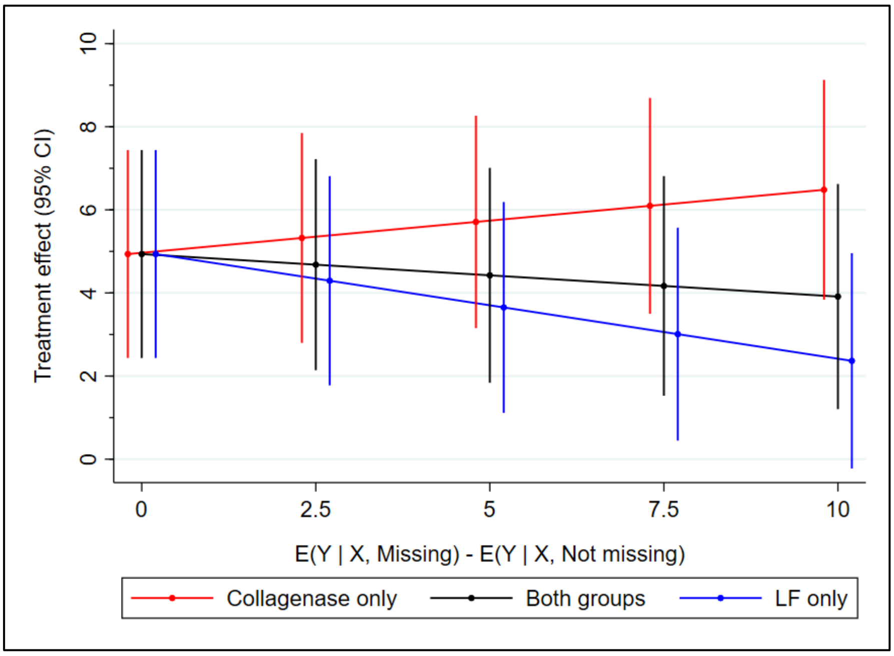

Figure S4: Point estimates and pointwise two-sided 95% confidence band for the difference in expected PEM score (positive values indicate greater benefit from LF). The red diamond denotes the non-inferiority margin for the primary endpoint. The 95% confidence intervals are not adjusted for multiplicity.

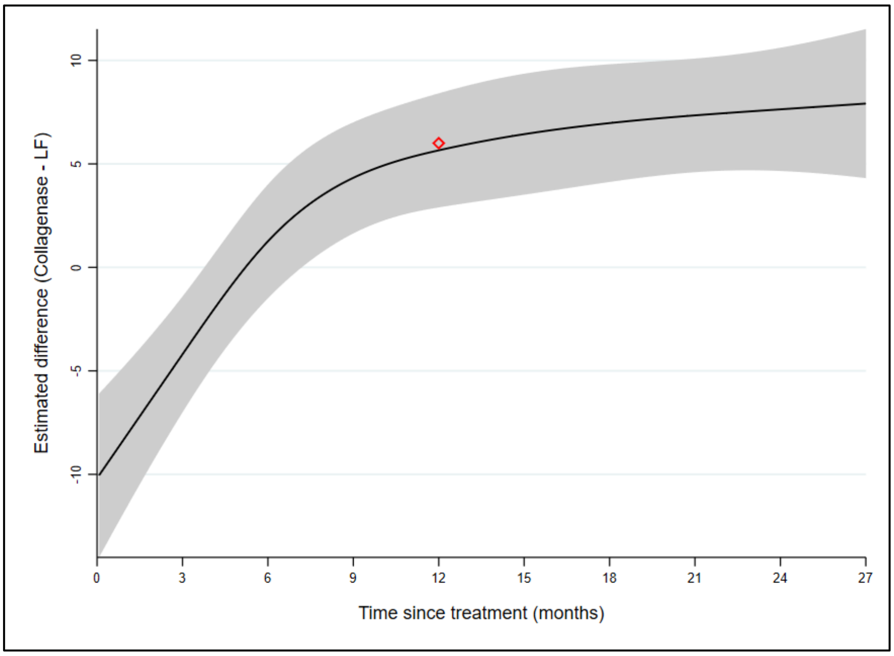

Figure S5: Point estimates and two-sided 95% confidence intervals for the difference (Collagenase - LF) in expected PEM score by baseline treatment preference subgroup and time point. The 95% confidence intervals are not adjusted for multiplicity

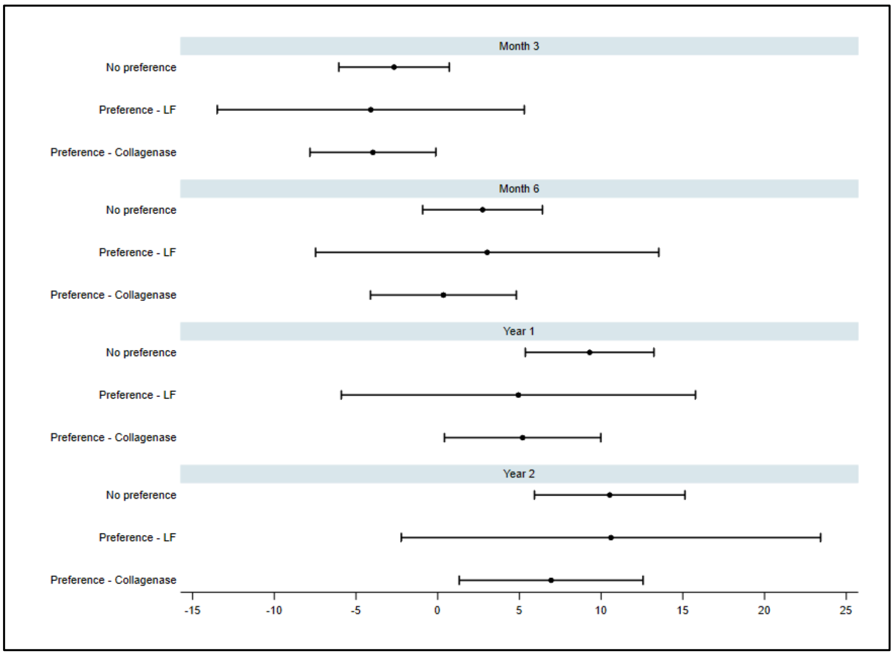

Figure S6: Point estimates and two-sided 95% confidence intervals for the difference (Collagenase - LF) in expected PEM score by study reference joint subgroup and time point. The 95% confidence intervals are not adjusted for multiplicity

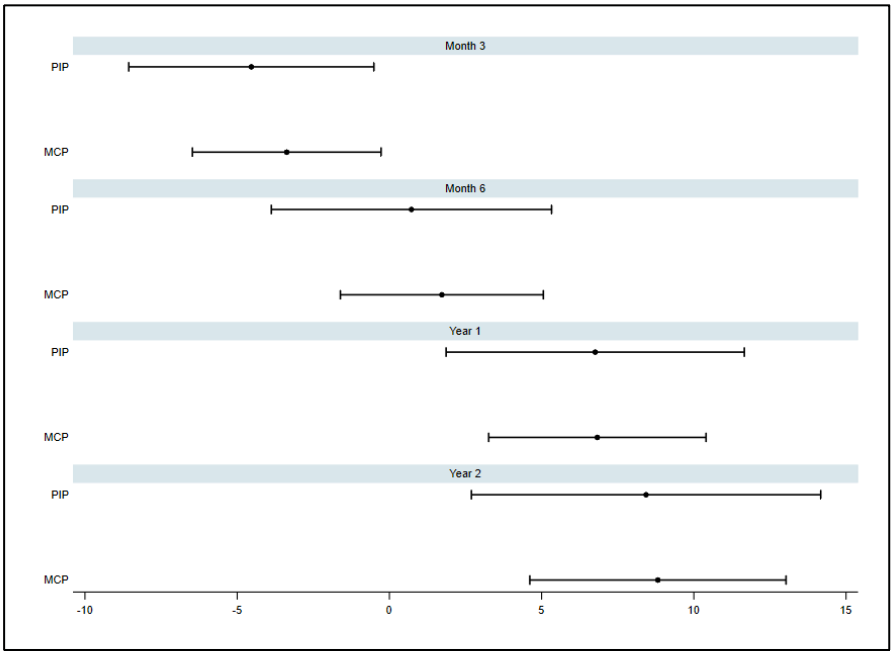

Figure S7: Mean score profiles over time stratified by treatment group for the joint measurement outcomes These estimates are based on the expected scores obtained from the pre-specified analysis models for each outcome. Unadjusted summary statistics are shown for each outcome at each time point in Table S4.

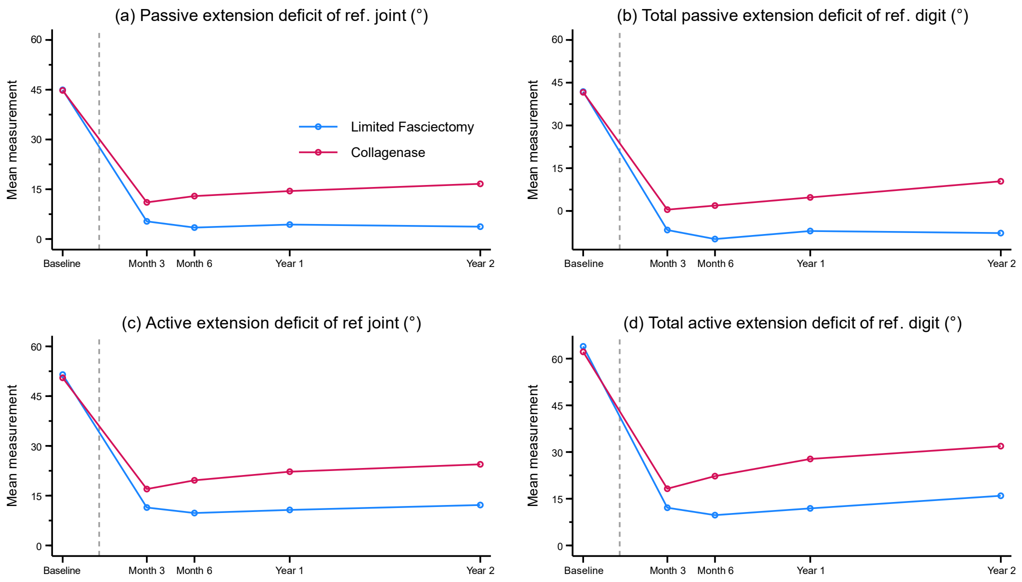

Table S1: DISC Outcomes and results summary table (* denotes the primary endpoint, PEM at 12 months post-treatment). The 95% confidence intervals are not adjusted for multiplicity.

Outcome measure	Brief description of outcome and analysis	Time point and sample size	Treatment effect (95% CI)[1]<br>D = Difference in means<br>OR = Odds Ratio<br>RD = Risk Difference<br>RR = Risk Ratio<br>HR = Hazard Ratio
Patient Evaluation Measure Hand Health Questionnaire (PEM) [2]	An 11 item, seven interval scale measuring disability associated with hand/wrist functionality/health. Scores range 0 – 100 with higher scores indicating a less favourable response.<br><br>Analysed using mixed effect generalised least squares. Estimated treatment effects (collagenase – LF) given in terms of difference in expected score	3 months<br>Collagenase N = 281<br>LF: N = 250	D = -3.91(-6.25 to -1.34)
		6 months<br>Collagenase N = 269<br>LF: N = 245	D = 1.43 (-1.22 to 4.07)
		1 Year*<br>Collagenase N = 284<br>LF: N = 250	D = 5.95 (3.12 to 8.77), $p = 0.49$ for a one tailed test of H0: $\delta > 6$
		2 Years<br>Collagenase N = 229<br>LF: N = 197	D = 7.53 (4.18 to 10.88)
Unite Rheumatologique des Affections de la Main (URAM)	A nine item, six interval disease specific disability scale. Scores range 0 – 45 with higher scores indicating a less favourable response.	3 months<br>Collagenase N = 282<br>LF: N = 250	D = 0.82 (-0.21 to 1.84)
		6 months	D = 2.12 (1.05 to 3.19)

	Analysed using mixed effect generalised least squares. Estimated treatment effects (collagenase – LF) given in terms of difference in expected score.<br><br>The MCID for URAM is variable between 1.5-10.5. [1, 2]	Collagenase N = 269<br><br>LF: N = 245<br><br>1 Year<br><br>Collagenase N = 282<br><br>LF: N = 247<br><br>2 Years<br><br>Collagenase N = 225<br><br>LF: N = 198	D = 3.42 (2.18 to 4.66)<br><br>D = 5.37 (3.85 to 6.88)
Michigan Hand Questionnaire (MHQ)	A validated, 63 item questionnaire featuring six domains: overall hand function; activities of daily living; work performance; pain; aesthetics; patient satisfaction with hand function. Scores range 0 – 100 with higher scores indicating a more favourable response.<br><br>Analysed using mixed effect generalised least squares. Estimated treatment effects given in terms of difference in expected score.<br><br>Previous studies suggest the minimum clinically important difference for the MHQ is around 1-2 points.[3] MCID for MHQ is 1.4 and is quoted by the systematic review.[1]	1 Year<br><br>Collagenase N = 273<br><br>LF: N = 236<br><br>2 Years<br><br>Collagenase N = 218<br><br>LF: N = 189	D = -4.69 (-7.27 to -2.12)<br><br>D = -6.71 (-9.60 to -3.82)
Single Assessment Numeric	A single, patient reported, question assessing overall functionality of the hand.	2 Weeks<br><br>Collagenase N = 287	D = 14.93 (11.66 to 18.19)

Evaluation (SANE)	Scores range 0 – 100 with higher scores indicating a more favourable response.<br><br>Analysed using mixed effect generalised least squares. Estimated treatment effects given in terms of difference in expected score	LF: N = 256<br><br>6 Weeks<br>Collagenase N = 277<br>LF: N = 236<br><br>3 Months<br>Collagenase N = 280<br>LF: N = 244<br><br>6 Months<br>Collagenase N = 267<br>LF: N = 244<br><br>1 Year<br>Collagenase N = 282<br>LF: N = 245<br><br>2 Years<br>Collagenase N = 222<br>LF: N = 196	D = 5.00 (2.29 to 7.70)<br><br>D = 1.07 (-1.64 to 3.78)<br><br>D = -3.28 (-5.96 to -0.59)<br><br>D = -4.93 (-7.63 to -2.22)<br><br>D = -8.65 (-11.80 to -5.50)
Overall hand assessment	A single question asking "*Overall, how are the problems now, with the hand in which you had treatment, compared to before?"*, with seven levels of response: (1) Cured, (2) Much better, (3) A little better, (4) The same, (5) A little worse, (6) Much worse, (7) Terrible.<br><br>Analysed using a proportional odds model.	1 Year<br>Collagenase N = 283 (68.6% reported hand was "cured" or "much better")<br>LF: N = 244 (88.1% reported hand was "cured" or "much better")	OR for reporting higher (i.e. "worse") category = 3.01 (2.15 to 4.23)<br>RD for reporting "cured" or "much better" = -17.1% (-22.3% to -11.9%)

	Estimated treatment effect given in terms of the odds ratio for reporting a higher (i.e. worse) category		
Passive extension deficit of reference joint	Passive extension deficit of the designated study reference joint (obtained using goniometry, larger values indicate greater passive extension deficit)<br><br>Analysed using mixed effect generalised least squares. Estimated treatment effects given in terms of difference in expected score	3 months<br>Collagenase N = 226<br>LF: N = 210<br>6 months<br>Collagenase N = 203<br>LF: N = 171<br>1 Year<br>Collagenase N = 172<br>LF: N = 148<br>2 Years<br>Collagenase N = 142<br>LF: N = 116	D = 5.73 (2.88 to 8.59)<br>D = 9.48 (6.19 to 12.77)<br>D = 10.10 (6.46 to 13.73)<br>D = 12.92 (8.38 to 17.46)
Total passive extension deficit of reference digit	The sum of the passive extension deficit measurements obtained for the two/three joints of the reference digit.<br><br>Analysed using mixed effect generalised least squares. Estimated treatment effects given in terms of difference in expected score	3 months<br>Collagenase N = 220<br>LF: N = 207<br>6 months<br>Collagenase N = 199<br>LF: N = 169<br>1 Year<br>Collagenase N = 169<br>LF: N = 145<br>2 Years	D = 7.15 (2.25 to 12.05)<br>D = 11.75 (6.29 to 17.21)<br>D = 11.79 (5.68 to 17.89)<br>D = 18.18 (10.48 to 25.87)

		Collagenase N = 141<br><br>LF: N = 116	
Recurrence	Binary. Recurrence was defined as an increase in passive extension deficit of the reference joint of ≥20° between 3- and 12-months post-treatment. If participants were missing 12-month passive extension measurements of the reference joint, but had relevant measurements at 24 months that indicated an increase in extension deficit of <20°, then these participants were assumed to have not had recurrence by 12 months. If participants could not have recurrence/non-recurrence assigned on the basis of their 12- or 24-month measurements, then recurrence was defined as an increase in passive extension deficit of the designated reference joint of ≥6° between 3- and 6-months post-treatment.<br><br>Analysed using mixed effect binary logistic regression. Estimated treatment effect given terms of the odds ratio for meeting the definition of recurrence outlined above	Up to 12 months<br><br>Collagenase N = 186 (17.2%)<br><br>LF: N = 159 (13.5%)	OR = 1.39 (0.74 to 2.63)<br><br>Marginal RD = 4.0% (-3.7% to 11.7%)

Active extension deficit of reference joint	Passive extension deficit of the designated study reference joint (obtained using goniometry and photography, larger values indicate greater active extension deficit)<br><br>Analysed using mixed effect generalised least squares. Estimated treatment effects given in terms of difference in expected score	3 months<br>Collagenase N = 249<br>LF: N = 224<br>6 months<br>Collagenase N = 222<br>LF: N = 196<br>1 Year<br>Collagenase N = 209<br>LF: N = 188<br>2 Years<br>Collagenase N = 181<br>LF: N = 162	D = 5.57 (3.02 to 8.12)<br><br>D = 9.86 (6.71 to 13.02)<br><br>D = 11.52 (8.13 to 14.91)<br><br>D = 12.26 (8.30 to 16.23)
Total active extension deficit of reference digit	The sum of the active extension deficit measurements obtained for the two/three joints of the reference digit.<br><br>Analysed using mixed effect generalised least squares. Estimated treatment effects given in terms of difference in expected score	3 months<br>Collagenase N = 248<br>LF: N = 224<br>6 months<br>Collagenase N = 222<br>LF: N = 195<br>1 Year<br>Collagenase N = 209<br>LF: N = 188<br>2 Years<br>Collagenase N = 181<br>LF: N = 162	D = 6.10 (2.08 to 10.11)<br><br>D = 12.53 (7.82 to 17.25)<br><br>D = 15.87 (10.53 to 21.22)<br><br>D = 15.95 (9.75 to 22.14)

Treatment complications	Worst severity of treatment complication experienced following trial treatment ranked using the following seven level scale: None, Very minor, Mild, Moderate, Severe, Devastating, Death<br><br>Analysed using a partial proportional odds model (effect of allocation unconstrained across cut points of the outcome). Estimated treatment effects given in terms of the odds ratios for experiencing a complication at least as bad as the severity specified.	Up to 2 years<br><br>Collagenase N = 326<br><br>LF: N = 295	Very minor or worse<br><br>OR = 1.27 (0.92 to 1.76)<br><br>Marginal RD = 5.6% (-2.0% to 13.2%)<br><br>Mild or worse<br><br>OR = 1.01 (0.72 to 1.42)<br><br>Marginal RD = 0.3% (-7.1% to 7.7%)<br><br>Moderate or worse<br><br>OR = 0.35 (0.13 to 0.91)<br><br>Approximate RR = 0.4 (0.1 to 0.9)<br><br>Marginal RD = -3.3% (-6.2% to -0.4%)<br><br>Severe or worse<br><br>OR undefined (all three events rated severe or worse occurred in the LF group)
Time to re-intervention	Time to first re-intervention on the reference digit using any of the following treatments: collagenase injection, Limited fasciectomy, percutaneous needle fasciotomy, dermofasciectomy.<br><br>Analysed using Cox proportional hazards model. Estimated treatment effects	Up to 2 years<br><br>Collagenase N = 149<br><br>LF: N = 178	HR = 4.73 (1.81 to 12.34)

	given in terms of the hazard ratio for re-intervention.		
EQ-5D-5L	Used to estimate QALYs as part of the health economic analyses	2 Weeks<br>Collagenase N = 281<br>LF: N = 253	N/A – not analysed as part of clinical effectiveness analyses
		6 Weeks<br>Collagenase N = 275<br>LF: N = 234	N/A – not analysed as part of clinical effectiveness analyses
		3 Months<br>Collagenase N = 278<br>LF: N = 249	N/A – not analysed as part of clinical effectiveness analyses
		6 Months<br>Collagenase N = 264<br>LF: N = 243	N/A – not analysed as part of clinical effectiveness analyses
		1 Year<br>Collagenase N = 281<br>LF: N = 247	N/A – not analysed as part of clinical effectiveness analyses
		2 Years<br>Collagenase N = 227<br>LF: N = 196	N/A – not analysed as part of clinical effectiveness analyses

[1] *95% CIs for these outcomes were not adjusted for multiplicity and should not be used in place of hypothesis testing*

[2] *PEM was selected based on a peer-reviewed funding process as the suitable primary outcome. PEM is a holistic hand disorder outcome measure which includes the capacity to capture the impact of treatment complications. It is similar to the Michigan Hand Questionnaire (MHQ) and the Disabilities of the Arm Shoulder and Hand (DASH).(4-6) In addition it provides a Patient Rated Experience Measure in section 1, assessment of pain, other symptoms, and disability in section 2, and a summary, including a Global Rating of Change in section 3. PEM is also being used in the ongoing NIHR-commissioned HAND-2 trial looking at Percutaneous Needle Fasciotomy vs Limited Fasciectomy for similar reasons. Its sensitivity compared to MHQ and DASH, also its ease of use has been reported on previously(4-6). We included the URAM which is a disorder specific questionnaire which explores the impact of contracture alone on activities of daily living; and the MHQ to ensure consistency of findings. The DISC trial was powered to detect a difference on all other PROMs, which were included as secondary outcomes.*

Table S2: Experience of surgeons delivering/supervising collagenase administration and limited fasciectomy surgery.

	**Patients receiving LF** (N = 287)	**Patients receiving Collagenase** (N = 331)
**Present during procedure, n (%)**		
Consultant surgeon*	277 (96.5)	325 (98.2)
Trainee surgeon*	7 (2.4)	6 (1.8)
Missing	3 (1.1)	0 (0.0)

* Consultants are akin to "attending surgeon" in the US and trainees are similar to "residents"

Table S3: Treatment effect estimates at each post-treatment time point obtained from univariate semi-parametric analyses (proportional odds model) of the available scores (boldface denotes the primary endpoint). The reported estimates are conditioned on the observed mean baseline PEM score and each level of reference joint type. The 95% confidence intervals are not adjusted for multiplicity.

	**Reference joint**	**Estimated difference (Collagenase - LF) in expected score (95% CI*)**
Month 3	MCP	-3.20 (-5.26 to -1.13)
	PIP	-3.92 (-6.44 to -1.40)
Month 6	MCP	1.82 (-0.32 to 3.96)
	PIP	2.24 (-0.40 to 4.89)
**Month 12**	**MCP**	**4.16 (1.91 to 6.40)**
	**PIP**	**5.49 (2.48 to 8.51)**
Month 24	MCP	7.46 (4.67 to 10.24)
	PIP	9.71 (6.02 to 13.39)

*Two-sided, Wald method, based on delta method standard errors

Table S4: Treatment effect estimates at 3, 6, 12 and 24 months post-treatment obtained from primary analysis model including additional baseline predictors of incomplete primary outcome data (boldface indicates primary endpoint).The 95% confidence intervals are not adjusted for multiplicity

	**Estimated difference (Collagenase - LF) (95% CI*)**
Month 3	-3.76 (-6.22 to -1.31)
Month 6	1.51 (-1.12 to 4.15)
**Month 12**	**6.02 (3.22 to 8.81)**
Month 24	7.38 (4.04 to 10.71)

*Two-sided, based on t-test with degrees of freedom calculated using the Kenward-Roger method

Table S5: Treatment effect estimates at 3, 6, 12 and 24 months post-treatment from primary analysis undertaken using multiply imputed data. The 95% confidence intervals are not adjusted for multiplicity

	**Estimated difference (Collagenase - LF) (95% CI*)**
Month 3	-3.32 (-5.69 to -0.95)
Month 6	2.15 (-0.35 to 4.64)
**Month 12**	**6.52 (3.89 to 9.15)**
Month 24	7.53 (4.49 to 10.57)

*Two-sided, Wald method, based on variance estimates obtained via Rubin's rules

Table S6: Treatment effect estimates at 3, 6, 12 and 24 months post-treatment obtained from an analysis of the primary outcome data conditioning on the pre-treatment PEM measurements (boldface denotes the primary endpoint). The 95% confidence intervals are not adjusted for multiplicity

	**Estimated difference (Collagenase - LF) (95% CI*)**
Month 3	-3.84 (-6.27 to -1.41)
Month 6	1.38 (-1.22 to 3.98)
**Month 12**	**5.87 (3.11 to 8.62)**
Month 24	7.47 (4.18 to 10.76)

*Two-sided, based on t-test with degrees of freedom calculated using the Kenward-Roger method

Table S7: Treatment effect estimates at 3, 6, 12 and 24 months post-treatment obtained from an analysis including only primary outcome data collected within the protocol specified follow-up windows (boldface denotes the primary endpoint). The 95% confidence intervals are not adjusted for multiplicity

	**Estimated difference (Collagenase - LF) (95% CI*)**
Month 3	-4.30 (-7.08 to -1.51)
Month 6	2.41 (-0.61 to 5.43)
**Month 12**	**6.09 (3.29 to 8.89)**
Month 24	8.33 (5.00 to 11.66)

*Two-sided, based on t-test with degrees of freedom calculated using the Kenward-Roger method

Table S8: Treatment effect estimates at 3, 6, 12 and 24 months post-treatment obtained from analysis including (variable) time from treatment as a continuous predictor (boldface denotes the primary endpoint). The 95% confidence intervals are not adjusted for multiplicity

	**Estimated difference (Collagenase - LF) (95% CI*)**
Month 3	-4.20 (-7.00 to -1.40)
Month 6	1.26 (-1.49 to 4.01)
**Month 12**	**5.66 (2.90 to 8.41)**
Month 24	7.64 (4.66 to 10.61)

*Two-sided, based on t-test with degrees of freedom calculated using the Kenward-Roger method

Table S9: Results from post-hoc per-protocol analysis requested during review (boldface denotes the primary endpoint). The 95% confidence intervals are not adjusted for multiplicity

	**Estimated difference (Collagenase - LF) (95% CI*)**
Month 3	-3.90 (-6.35 to -1.45)
Month 6	1.43 (-1.21 to 4.07)
**Month 12**	**6.38 (3.59 to 9.18)**
Month 24	7.82 (4.52 to 11.12)

*Two-sided, based on t-test with degrees of freedom calculated using the Kenward-Roger method

Table S10: Baseline treatment preferences – participants with available primary outcome data only

	**LF** N = 285	**Collagenase** N = 314	**Total** N = 599
**Treatment preference, n (%)**			
Collagenase injection	108 (37.9)	135 (43.0)	243 (40.6)
Surgical intervention	25 (8.8)	16 (5.1)	41 (6.8)
No preference	148 (51.9)	158 (50.3)	306 (51.1)
Missing	4 (1.4)	5 (1.6)	9 (1.5)

Table S11: Designated study reference digit – participants with available primary outcome data only

	**LF**<br>N = 285	**Collagenase**<br>N = 314	**Total**<br>N = 599
**Study reference joint, n (%)**			
MCP	172 (60.4)	204 (65.0)	376 (62.8)
PIP	113 (39.6)	110 (35.0)	223 (37.2)

Table S12: Treatment effect estimates by baseline treatment preference subgroup and time point (includes only participants with non-missing baseline treatment preferences. The 95% confidence intervals are not adjusted for multiplicity

**Time point**	**Baseline treatment preference**	**Estimated difference (Collagenase - LF) (95% CI)**
Month 3	Preference - collagenase	-3.96 (-7.81 to -0.11)
	Preference - LF	-4.09 (-13.49 to 5.30)
	No preference	-2.67 (-6.05 to 0.71)
Month 6	Preference - collagenase	0.35 (-4.11 to 4.81)
	Preference - LF	3.03 (-7.47 to 13.53)
	No preference	2.75 (-0.92 to 6.41)
Month 12	Preference - collagenase	5.20 (0.41 to 9.98)
	Preference - LF	4.94 (-5.90 to 15.77)
	No preference	9.30 (5.36 to 13.23)
Month 24	Preference - collagenase	6.94 (1.32 to 12.56)
	Preference - LF	10.60 (-2.23 to 23.43)
	No preference	10.53 (5.92 to 15.13)

Table S13: Treatment effect estimates by baseline reference joint subgroup and time point. The 95% confidence intervals are not adjusted for multiplicity

**Time point**	**Study reference joint**	**Estimated difference (Collagenase - LF) (95% CI)**
Month 3	MCP	-3.37 (-6.47 to -0.27)
	PIP	-4.54 (-8.56 to -0.51)
Month 6	MCP	1.72 (-1.61 to 5.06)
	PIP	0.73 (-3.88 to 5.33)
Month 12	MCP	6.83 (3.26 to 10.40)
	PIP	6.76 (1.86 to 11.66)
Month 24	MCP	8.82 (4.61 to 13.03)
	PIP	8.43 (2.69 to 14.17)

Table S14: Summaries of patient reported outcome scores by time point and allocation (all available cases).

	**LF** N = 336	**Collagenase** N = 336	**Total** N = 672
**Patient Evaluation Measure (PEM)**			
**Baseline**			
N	333	334	667
Mean (SD)	34.1 (19.7)	34.2 (20.2)	34.2 (19.9)
Median (Q1, Q3)	31.8 (18.2, 48.5)	31.8 (18.2, 47.0)	31.8 (18.2, 48.5)
Min, Max	0.0, 87.9	0.0, 93.9	0.0, 93.9
**Pre-treatment**			
N	274	322	596
Mean (SD)	34.9 (19.0)	35.5 (20.2)	35.2 (19.7)
Median (Q1, Q3)	34.8 (19.7, 48.5)	33.3 (19.7, 50.0)	34.1 (19.7, 48.5)
Min, Max	0.0, 84.8	0.0, 95.5	0.0, 95.5
**Month 3**			
N	250	281	531
Mean (SD)	16.2 (16.6)	12.7 (14.3)	14.3 (15.5)
Median (Q1, Q3)	10.6 (3.0, 22.7)	9.1 (1.5, 19.7)	9.1 (3.0, 21.2)
Min, Max	0.0, 83.3	0.0, 81.8	0.0, 83.3
**Month 6**			
N	245	269	514
Mean (SD)	13.0 (16.4)	14.6 (16.9)	13.9 (16.7)
Median (Q1, Q3)	7.6 (1.5, 16.7)	9.1 (1.5, 21.2)	8.3 (1.5, 19.7)
Min, Max	0.0, 86.4	0.0, 90.9	0.0, 90.9
**Month 12**			
N	250	284	534
Mean (SD)	12.4 (16.3)	16.8 (19.3)	14.7 (18.1)
Median (Q1, Q3)	6.1 (0.0, 18.2)	9.1 (1.5, 24.2)	7.6 (1.5, 22.7)
Min, Max	0.0, 87.9	0.0, 95.0	0.0, 95.0
**Month 24**			
N	197	229	426
Mean (SD)	12.2 (18.0)	19.2 (20.7)	15.9 (19.8)
Median (Q1, Q3)	4.5 (0.0, 15.2)	12.1 (3.0, 30.0)	7.6 (1.5, 22.7)
Min, Max	0.0, 84.8	0.0, 95.5	0.0, 95.5
**Unité Rhumatologique des Affections de la Main (URAM)**			
**Baseline**			
N	335	333	668
Mean (SD)	17.2 (9.5)	17.0 (9.3)	17.1 (9.4)
Median (Q1, Q3)	16.9 (10.0, 24.0)	16.0 (10.0, 23.0)	16.0 (10.0, 23.0)
Min, Max	0.0, 43.0	0.0, 44.0	0.0, 44.0
**Month 3**			
N	250	282	532

Mean (SD)	3.8 (6.1)	4.8 (6.7)	4.3 (6.5)
Median (Q1, Q3)	1.0 (0.0, 6.0)	2.0 (0.0, 7.0)	1.0 (0.0, 6.0)
Min, Max	0.0, 32.0	0.0, 38.0	0.0, 38.0
**Month 6**			
N	245	269	514
Mean (SD)	3.2 (5.6)	5.5 (7.1)	4.4 (6.5)
Median (Q1, Q3)	1.0 (0.0, 4.0)	2.0 (0.0, 9.0)	1.0 (0.0, 7.0)
Min, Max	0.0, 35.0	0.0, 40.0	0.0, 40.0
**Month 12**			
N	247	282	529
Mean (SD)	3.9 (6.7)	6.6 (8.5)	5.4 (7.8)
Median (Q1, Q3)	1.0 (0.0, 6.0)	3.0 (0.0, 10.0)	2.0 (0.0, 8.0)
Min, Max	0.0, 45.0	0.0, 42.0	0.0, 45.0
**Month 24**			
N	198	225	423
Mean (SD)	3.9 (7.2)	8.8 (9.7)	6.5 (8.9)
Median (Q1, Q3)	0.0 (0.0, 4.0)	6.0 (0.0, 14.0)	2.0 (0.0, 10.0)
Min, Max	0.0, 39.0	0.0, 42.0	0.0, 42.0
**Michigan Hand Questionnaire (MHQ)**			
**Baseline**			
N	328	326	654
Mean (SD)	67.5 (17.7)	67.6 (17.1)	67.6 (17.4)
Median (Q1, Q3)	70.4 (55.2, 81.1)	70.1 (56.4, 81.1)	70.3 (55.6, 81.1)
Min, Max	21.3, 100.0	15.5, 99.0	15.5, 100.0
**Month 12**			
N	236	273	509
Mean (SD)	86.5 (15.1)	81.8 (17.9)	84.0 (16.8)
Median (Q1, Q3)	91.6 (78.7, 98.8)	87.5 (70.3, 96.7)	88.8 (74.8, 97.9)
Min, Max	26.2, 100.0	14.5, 100.0	14.5, 100.0
**Month 24**			
N	189	218	407
Mean (SD)	87.2 (15.8)	79.9 (18.5)	83.3 (17.7)
Median (Q1, Q3)	93.2 (83.3, 99.0)	86.1 (67.1, 95.2)	89.0 (73.7, 97.9)
Min, Max	30.2, 100.0	22.4, 100.0	22.4, 100.0
**Single Assessment Numeric Evaluation (SANE)**			
**Baseline**			
N	328	334	662
Mean (SD)	61.6 (21.7)	62.0 (21.8)	61.8 (21.7)
Median (Q1, Q3)	65.0 (49.0, 80.0)	65.0 (49.0, 80.0)	65.0 (49.0, 80.0)
Min, Max	10.0, 100.0	0.0, 100.0	0.0, 100.0
**Week 2**			
N	256	287	543
Mean (SD)	62.5 (22.1)	78.1 (17.7)	70.7 (21.4)
Median (Q1, Q3)	65.0 (50.0, 80.0)	83.0 (70.0, 90.0)	75.0 (60.0, 90.0)
Min, Max	0.0, 100.0	4.0, 100.0	0.0, 100.0
**Week 6**			
N	236	277	513

Mean (SD)	80.7 (16.1)	86.0 (16.3)	83.6 (16.4)
Median (Q1, Q3)	85.0 (75.0, 91.0)	90.0 (80.0, 95.0)	90.0 (80.0, 95.0)
Min, Max	10.0, 100.0	4.0, 100.0	4.0, 100.0
**Month 3**			
N	244	280	524
Mean (SD)	86.5 (16.1)	87.2 (16.5)	86.9 (16.3)
Median (Q1, Q3)	90.0 (80.0, 97.0)	91.0 (83.0, 98.0)	90.0 (80.0, 98.0)
Min, Max	8.0, 100.0	5.0, 100.0	5.0, 100.0
**Month 6**			
N	244	267	511
Mean (SD)	89.1 (14.3)	85.8 (16.9)	87.4 (15.8)
Median (Q1, Q3)	95.0 (85.5, 98.0)	90.0 (80.0, 98.0)	93.0 (80.0, 98.0)
Min, Max	20.0, 100.0	5.0, 100.0	5.0, 100.0
**Month 12**			
N	245	282	527
Mean (SD)	88.7 (14.7)	84.8 (17.8)	86.6 (16.5)
Median (Q1, Q3)	95.0 (85.0, 99.0)	90.0 (80.0, 98.0)	91.0 (80.0, 98.0)
Min, Max	10.0, 100.0	9.0, 100.0	9.0, 100.0
**Month 24**			
N	196	222	418
Mean (SD)	89.0 (14.8)	81.4 (19.0)	85.0 (17.5)
Median (Q1, Q3)	95.0 (85.0, 99.0)	89.5 (73.0, 95.0)	90.0 (79.0, 97.0)
Min, Max	20.0, 100.0	12.0, 100.0	12.0, 100.0

Table S15: Summaries of joint measurement outcomes by time point and allocation (all available cases)

	**LF**<br>N = 336	**Collagenase**<br>N = 336	**Total**<br>N = 672
**Passive extension deficit of reference joint (°)**			
**Baseline**			
N	288	318	606
Mean (SD)	46.0 (16.7)	45.6 (17.2)	45.8 (17.0)
Median (Q1, Q3)	44.7 (34.0, 59.0)	45.2 (32.0, 58.3)	44.7 (32.7, 58.7)
Min, Max	0.0, 90.0	-10.0, 84.7	-10.0, 90.0
**Pre-treatment**			
N	265	309	574
Mean (SD)	49.5 (17.8)	48.3 (17.1)	48.9 (17.4)
Median (Q1, Q3)	50.0 (38.0, 62.0)	46.0 (38.0, 60.0)	48.0 (38.0, 60.0)
Min, Max	0.0, 91.0	-10.0, 92.0	-10.0, 92.0
**Month 3**			
N	210	226	436
Mean (SD)	5.6 (19.9)	9.5 (21.0)	7.6 (20.5)
Median (Q1, Q3)	0.7 (-7.3, 18.3)	2.7 (-3.3, 23.3)	1.7 (-5.2, 20.0)
Min, Max	-35.0, 70.7	-42.7, 74.0	-42.7, 74.0
**Month 6**			

N	171	203	374
Mean (SD)	5.1 (22.8)	11.4 (22.5)	8.5 (22.8)
Median (Q1, Q3)	0.0 (-10.0, 16.0)	6.0 (-0.7, 25.0)	2.0 (-5.0, 20.7)
Min, Max	-30.7, 78.0	-48.0, 80.0	-48.0, 80.0
**Month 12**			
N	148	172	320
Mean (SD)	4.0 (23.6)	13.0 (25.3)	8.8 (24.9)
Median (Q1, Q3)	0.0 (-11.3, 18.7)	7.7 (-5.2, 29.3)	1.0 (-7.7, 25.5)
Min, Max	-72.0, 73.3	-38.0, 78.7	-72.0, 78.7
**Month 24**			
N	116	142	258
Mean (SD)	5.4 (26.6)	16.5 (27.9)	11.5 (27.9)
Median (Q1, Q3)	0.0 (-12.3, 18.8)	11.0 (-3.3, 38.7)	6.0 (-9.3, 30.7)
Min, Max	-40.0, 90.0	-50.0, 88.7	-50.0, 90.0
**Total passive extension deficit of reference digit (°)**			
**Baseline**			
N	271	299	570
Mean (SD)	43.5 (35.9)	42.3 (35.6)	42.9 (35.8)
Median (Q1, Q3)	38.0 (20.0, 64.0)	39.3 (19.3, 64.0)	38.7 (20.0, 64.0)
Min, Max	-91.7, 175.0	-65.0, 144.0	-91.7, 175.0
**Pre-treatment**			
N	252	300	552
Mean (SD)	52.6 (36.5)	49.4 (36.9)	50.8 (36.7)
Median (Q1, Q3)	50.0 (28.0, 74.0)	46.0 (24.5, 71.0)	48.0 (26.0, 74.0)
Min, Max	-42.0, 168.0	-44.0, 186.0	-44.0, 186.0
**Month 3**			
N	207	220	427
Mean (SD)	-6.2 (29.7)	-1.5 (30.3)	-3.7 (30.1)
Median (Q1, Q3)	-7.3 (-27.3, 10.0)	-1.0 (-20.7, 15.5)	-3.3 (-23.3, 14.0)
Min, Max	-83.7, 98.0	-78.0, 102.0	-83.7, 102.0
**Month 6**			
N	169	199	368
Mean (SD)	-7.0 (31.9)	-0.7 (31.0)	-3.6 (31.5)
Median (Q1, Q3)	-10.0 (-28.7, 12.0)	0.0 (-18.7, 17.3)	-4.7 (-26.0, 15.0)
Min, Max	-73.3, 118.7	-87.3, 123.3	-87.3, 123.3
**Month 12**			
N	145	169	314
Mean (SD)	-8.7 (30.0)	3.0 (34.0)	-2.4 (32.7)
Median (Q1, Q3)	-5.0 (-32.0, 11.3)	0.0 (-19.3, 20.0)	-1.3 (-26.0, 17.3)
Min, Max	-78.7, 60.0	-75.0, 130.7	-78.7, 130.7
**Month 24**			
N	116	141	257
Mean (SD)	-8.3 (33.6)	9.0 (38.9)	1.2 (37.5)
Median (Q1, Q3)	-9.3 (-34.8, 10.0)	10.0 (-15.0, 28.0)	0.0 (-25.3, 22.7)
Min, Max	-77.3, 90.0	-75.7, 145.3	-77.3, 145.3
**Active extension deficit of reference joint (°)**			
**Baseline**			
N	292	324	616

Mean (SD)	52.5 (15.5)	51.4 (16.6)	51.9 (16.1)
Median (Q1, Q3)	51.7 (40.0, 63.3)	50.7 (39.2, 62.7)	51.3 (40.0, 63.3)
Min, Max	10.0, 91.3	2.3, 90.7	2.3, 91.3
**Pre-treatment**			
N	282	318	600
Mean (SD)	54.1 (16.6)	52.8 (16.8)	53.4 (16.7)
Median (Q1, Q3)	54.0 (40.0, 66.0)	50.0 (42.0, 64.0)	50.0 (42.0, 65.0)
Min, Max	10.0, 100.0	8.0, 91.0	8.0, 100.0
**Month 3**			
N	224	249	473
Mean (SD)	12.1 (19.0)	15.4 (19.7)	13.8 (19.4)
Median (Q1, Q3)	5.3 (0.0, 23.3)	10.7 (0.0, 27.3)	9.3 (0.0, 26.0)
Min, Max	-26.7, 77.0	-34.0, 71.3	-34.0, 77.0
**Month 6**			
N	196	222	418
Mean (SD)	10.9 (21.5)	18.1 (22.8)	14.7 (22.5)
Median (Q1, Q3)	4.7 (-0.7, 20.0)	12.7 (0.0, 32.0)	9.7 (0.0, 26.7)
Min, Max	-28.0, 81.0	-48.7, 86.7	-48.7, 86.7
**Month 12**			
N	188	209	397
Mean (SD)	10.7 (22.9)	20.0 (24.5)	15.6 (24.2)
Median (Q1, Q3)	5.3 (0.0, 19.3)	14.7 (1.3, 35.0)	9.0 (0.0, 30.7)
Min, Max	-74.7, 87.5	-40.0, 86.0	-74.7, 87.5
**Month 24**			
N	162	181	343
Mean (SD)	12.1 (23.7)	22.7 (26.0)	17.7 (25.5)
Median (Q1, Q3)	5.2 (0.0, 22.3)	19.0 (3.0, 41.5)	10.0 (0.0, 34.0)
Min, Max	-29.0, 90.0	-50.0, 90.0	-50.0, 90.0
**Total active extension deficit of reference digit (°)**			
**Baseline**			
N	286	322	608
Mean (SD)	64.6 (30.8)	62.4 (31.7)	63.4 (31.3)
Median (Q1, Q3)	59.4 (40.0, 87.3)	56.8 (40.0, 80.0)	58.7 (40.0, 83.5)
Min, Max	4.7, 151.7	0.0, 160.0	0.0, 160.0
**Pre-treatment**			
N	275	317	592
Mean (SD)	69.2 (33.8)	65.5 (33.2)	67.2 (33.5)
Median (Q1, Q3)	62.0 (44.0, 90.0)	60.0 (42.0, 82.0)	60.0 (42.0, 87.0)
Min, Max	4.0, 178.0	-3.5, 190.0	-3.5, 190.0
**Month 3**			
N	224	248	472
Mean (SD)	11.7 (26.2)	15.7 (25.4)	13.8 (25.8)
Median (Q1, Q3)	8.5 (-4.3, 28.0)	14.0 (0.0, 31.0)	11.5 (-2.2, 30.0)
Min, Max	-56.0, 90.0	-50.0, 100.0	-56.0, 100.0
**Month 6**			
N	195	222	417
Mean (SD)	10.7 (27.8)	19.1 (28.1)	15.1 (28.2)
Median (Q1, Q3)	7.0 (-6.7, 27.3)	15.7 (0.0, 34.0)	11.7 (-2.0, 30.7)
Min, Max	-54.0, 140.0	-63.3, 130.0	-63.3, 140.0
**Month 12**			

N	188	209	397
Mean (SD)	11.4 (25.9)	25.4 (33.2)	18.7 (30.7)
Median (Q1, Q3)	10.0 (-3.0, 29.2)	18.5 (3.5, 41.5)	13.3 (0.0, 34.0)
Min, Max	-64.0, 120.0	-65.3, 152.7	-65.3, 152.7
**Month 24**			
N	162	181	343
Mean (SD)	14.1 (31.2)	30.3 (35.2)	22.7 (34.3)
Median (Q1, Q3)	10.0 (-4.7, 30.0)	25.0 (8.0, 50.0)	16.0 (0.0, 42.0)
Min, Max	-49.3, 183.0	-62.0, 180.0	-62.0, 183.0

Table 16: Details of treatment complications and adverse events‡ according to treatment allocation.

**Classification of complications/adverse events‡**		
	**Limited fasciectomy**	**Collagenase**
	**N = 295**	**N = 326**
**Pain, swelling or stiffness**		
Pain, n (%*)	16 (5.4)	47 (14.4)
Pain, swelling tenderness, n (%*)	12 (4.1)	29 (8.9)
Pain, stiffness, n (%*)	28 (9.5)	4 (1.2)
Joint swelling, n (%*)	3 (1.0)	9 (2.8)
Joint pain (arthralgia), n (%*)	0 (0.0)	5 (1.5)
**Skin, scar or wound related (excluding wound infection)**		
Retained suture, n (%*)	1 (0.3)	0 (0.0)
Scar problems, n (%*)	21 (7.1)	2 (0.6)
Skin laceration, n (%*)	2 (0.7)	59 (18.1)
Wound problems, n (%*)	15 (5.1)	0 (0.0)
Wound (infection), n (%*)	6 (2.0)	0 (0.0)
**Nerve related**		

Nerve paresthesia, n (%*)	19 (6.4)	12 (3.7)
Nerve dysesthesia, n (%*)	6 (2.0)	5 (1.5)
Nerve hypoesthesia, n (%*)	9 (3.1)	3 (0.9)
Nerve injury, n (%*)	10 (3.4)	2 (0.6)
**Circulation or bleeding related**		
Ecchymosis, n (%*)	3 (1.0)	31 (9.5)
Erythema, n (%*)	1 (0.3)	4 (1.2)
Blood blister, n (%*)	1 (0.3)	19 (5.8)
Bleeding, n (%*)	8 (2.7)	2 (0.6)
Raynauds, n (%*)	5 (1.7)	2 (0.6)
**Significant Events**		
Amputation, n (%*)	1 (0.3)	0 (0.0)
Complex Regional Pain Syndrome - algodystrophy, n (%*)	2 (0.7)	0 (0.0)
**Systemic Events**		
Lymphangitis, n (%*)	0 (0.0)	2 (0.6)
Lymphadenopathy, n (%*)	0 (0.0)	3 (0.9)
**Other Events**		
Other, n (%*) ¶	4 (1.4)	6 (1.8)
Instability, n (%*)	0 (0.0)	1 (0.3)

*Of total number of participants (by arm or overall)

‡ Complications and hand specific events not related to treatment were considered adverse events.

¶ Other events for collagenase patients: Cubital tunnel (n=3); Dizziness (n=1); Headache (n=1); Nausea (n=1);Pruritis (n=3). Other events for Limited fasciectomy patients: Delayed discharge (n=1); Carpal tunnel (n=1), Cubital tunnel (n=1); Dizziness (n=1); Pruritis (n=1).

Table S17: Details of treatment complications graded as “Severe” or “Moderate” in severity.

ID (Event)	Allocation	Treatment received	Grade	Description
1	Collagenase	Collagenase	Moderate	Large skin laceration (Larger than 2cm)
2	LF	LF	Severe	Left little finger amputation. Flexion at the PIPJ was affecting function and use of hand. Now happy with function in hand post surgery.
3	LF	LF	Moderate	Stiffening, hypoesthesia and pain in finger. Suspected trapped nerve in scar tissue.
4	LF	LF	Moderate	Numbness at base of finger following procedure.
5	Collagenase	Collagenase	Moderate	Pain in right hand that is getting progressively worse. Patient struggles to grip especially in the morning time; but this does ease off in the course of the day
6	LF	LF	Moderate	Paresthesia & numbness of ulnar digital nerve. Not improved at six months
7	LF	LF	Moderate	Patient reported numbness to finger. No further documentation in clinic letters or medical notes.
8	LF	LF	Moderate	Delayed healing identified at three month follow up - small open area at the PIP joint crease which is still damp
9	LF	LF	Moderate	Scar tissue and stiffness at 3 month follow up.
10 (1)	LF	LF	Moderate	Stiffness reported in reference digit associated with scar tissue at 3 months
10 (2)	LF	LF	Moderate	Minimal to mild swelling on reference joint at 3 month follow up.
11	LF	LF	Severe	Post fasciectomy participant has had a significant swelling to hand and digits of left hand. This is particularly painful in MCP and PIP joints of 3rd,4th and 5th finger. Swelling has caused stiffness to all associated joints and is struggling with flexion range of movement actively due to the above. It is improving very slowly since initial onset
12	Collagenase	Collagenase	Moderate	Patient attended 3 month review. The MCP joint contracture has been corrected but he has developed instability of this joint as a result of releasing the chronic flexion contracture he had
13	LF	LF	Moderate	During the intervention surgery on the study ref digit, the ulnar digital nerve divided and required repair during the procedure
14	Collagenase	Collagenase	Moderate	Numbness present on RHS little finger at 6 month follow up
15	LF	LF	Severe	Difficulty with sensation in finger 2nd opinion sought with hand surgeon – complex regional pain syndrome discussed.
16	Collagenase	Collagenase	Moderate	Hypoaesthesia
17	LF	LF	Moderate	The participant reported numbness at the tip of the reference finger.
18	LF	LF	Moderate	Patient had operation August on study reference digit and to ring finger at same time. Accidental division of ulna digital nerve to ring (non-study reference) finger repaired at the same time. No issues after. Discharges to physiotherapy. Had wash out, incision and drainage to ring finger by on-call colleague. Admitted for IV antibiotics and IoD and discharged on the same day
19	Collagenase	Collagenase	Moderate	Cold intolerance with skin colour changes in response to cold (white/purple discolouration). This affects the whole right hand since injection but the right little finger the most
20 (1)	LF	LF	Moderate	Burning type scar pain causing significant discomfort.
20 (2)	LF	LF	Moderate	Dyasthesia following surgery. Altered sensation in fingertip right little finger + numbness around scar
21	LF	LF	Moderate	Swelling and stiffness of finger. Hypersensitivity and pain at scar site.

Table S18: Representativeness of DISC study population

**Disease under investigation**	Dupuytren’s disease patients with a contracture requiring intervention
**Special considerations related to**	
**Age**	The prevalence of Dupuytren's disease increases with age. Incidence is highest in men older than 50 years of age (mean age 55 years) and in women older than 60 years of age. [4, 5]<br><br>The need for intervention due to a contracture is subject to multiple factors. [6] There is an interval, probably several years after onset, before the contracture becomes severe enough to merit treatment.
**Sex and gender**	Dupuytren's disease is up to six times more common in men than in women in Western countries (i.e. Europe and North America). [5, 7]. More specifically, the UK has a ratio of 3:1 [4].
**Ethnicity and geography**	Dupuytren's disease is most often seen in people of northern European descent.<br><br>In most studies, individuals in northern Scotland, Iceland, and Norway have the highest prevalence of Dupuytren's contracture. [7, 8]<br><br>When looked at across multi-ethnic groups in the US, the disease has highest prevalence among White Caucasians. [9]

	UK-specific data by ethnicity has not been published. However, its demographics, including the presence of Nordic/Viking heritage in the population and socio-economic spread would suggest that it is similar to other northern European countries, with the disease occurring primarily in the White male population.
**Overall representativeness of this trial**	The participants in the DISC trial were all from the United Kingdom. The age distribution for the onset of Dupuytren's disease in the sample maps to published epidemiological studies. Participants were recruited based on an assessment of need for intervention due to moderate contracture. The ratio of men to women in the sample (4:1) reflects the literature for this disorder. The sample is overwhelmingly White Caucasian as would be expected for Dupuytren's Disease. The DISC trial results would be applicable to patient populations in Western countries.

References

1. Rodrigues JN BG, Ball C, Zhang W, Giele H, Hobby J, Pratt AL, Davis T. Surgery for Dupuytren's contracture of the fingers. *Cochrane Database of Systematic Reviews* 2015. DOI: https://doi.org/10.1002/14651858.CD010143.pub2.
2. Hensler S., Wehrli M., Herren DB., et al. Measurement properties of the German Unite Rhumatologique des Affections de la Main (URAM) scale in patients treated for Dupuytren's disease. *Hand Surgery and Rehabilitation* 2020; 39: 568-574. DOI: https://doi.org/10.1016/j.hansur.2020.05.008.
3. Bradet-Levesque I., Audet J., Roy JS., et al. Measuring functional outcome in Dupuytren's disease: A systematic review of patient-reported outcome measures. *J Hand Ther* 2021: S0894-1130(0821)00057-00050. DOI: https://doi.org/10.1016/j.jht.2021.04.010.
4. Geoghegan L, Man J, Jain A, et al. Factors Associated with the Development, Progression, and Outcome of Dupuytren Disease Treatment: A Systematic Review. *Plastic and Reconstructive Surgery* 2021; 148: 753e-763e. DOI: https://doi.org/10.1097/PRS.0000000000008420
5. Lanting R., Broekstra DC., Werker PMN., et al. A systematic review and meta-analysis on the prevalence of Dupuytren disease in the general population of Western countries. *Plast Reconstr Surg* 2014; 133: 593-603. DOI: https://doi.org/10.1097/01.prs.0000438455.37604.0f.
6. Dias JJ. The Epidemiology of Surgical Intervention for Dupuytren Contracture in England. In: Werker PMN., Dias JJ., Eaton C., et al. (eds) *Dupuytren Disease and Related Diseases- The Cutting Edge*. Switzerland: Springer, 2016.
7. NICE. Dupuytren's disease: How common is it? https://cks.nice.org.uk/topics/dupuytrens-disease/background-information/prevalence/#:~:text=Diagnosis-,How%20common%20is%20it%3F,thought%20to%20have%20Dupuytren's%20disease (2022, 22.08.2023).
8. Gudmundsson KG, Arngrímsson R, Sigfússon N, et al. Epidemiology of Dupuytren's disease: Clinical, serological, and social assessment. The Reykjavik Study. *Journal of Clinical Epidemiology* 2000; 53: 291-296. DOI: https://doi.org/10.1016/S0895-4356(99)00145-6.
9. Saboeiro A, Pokorny J, Shehadi S, et al. Racial Distribution of Dupuytren's Disease in Department of Veterans Affairs Patients. *Plast Reconstr Surg* 2000; 106: 71-75.
